# EPHA3, upregulated in high-risk histological subtypes of gastrointestinal stromal tumor, promotes GIST progression

**DOI:** 10.2478/raon-2026-0042

**Published:** 2026-09-07

**Authors:** Han Wei, Jia Zhao, Hongmei Wang, Lihong Guo, Yanhong Liu, Chuntao Wang, Liang Yang, Zhi Qi, Wencong Tian, Lei Cao, Yang Gao

**Affiliations:** 1 Department of Molecular Pharmacology, School of Medicine, Nankai University; Tianjin, 300071, China; 2 Department of General Surgery, Tianjin Union Medical Center, The First Affiliated Hospital of Nankai University, Nankai University, Tianjin, 300122, China; 3 Institute of Digestive Disease, Shengli Oilfield Central Hospital, Dongying, 257000, China

**Keywords:** EPHA3, gastrointestinal stromal tumors (GISTs), histological subtype, epithelial–mesenchymal transition (EMT)

## Abstract

**Background:**

To explore the expression pattern and functional role of EPH receptor A3 (EPHA3) in high-risk gastrointestinal stromal tumors (GISTs) and its potential relevance to tumor progression.

**Materials and methods:**

Immunohistochemical (IHC) analysis was performed to evaluate EPHA3 expression across a pilot cohort of clinical GIST specimens with different risk stratifications. The biological functions of EPHA3 *in vitro* were assessed using (5-ethynyl-2’-deoxyuridine, EdU) assay, colony formation, apoptosis, wound-healing, and transwell assays in EPHA3-knockout GIST-430 cells. Furthermore, RNA sequencing (RNA-seq) analysis was utilized to identify differentially expressed genes and associated signaling pathways following EPHA3 knockout.

**Results:**

EPHA3 expression was found to be elevated in high-risk GIST specimens compared with intermediate-risk cases. Loss-of-function assays demonstrated that EPHA3 knockout attenuated cell proliferation and migration while promoting apoptosis *in vitro*. Pathway enrichment analysis suggested that EPHA3 expression is associated with cell adhesion, extracellular matrix interaction, and mesenchymal-related phenotypic remodeling.

**Conclusions:**

Our findings suggest that EPHA3 is upregulated in high-risk GIST and contributes to aggressive cellular behavior *in vitro*. These preliminary data indicate that EPHA3 may play a role in the malignant progression of GIST, although its clinical utility as a biomarker requires further validation in larger cohorts.

## Introduction

Gastrointestinal stromal tumors (GISTs), the most frequent mesenchymal tumors in the gastrointestinal system, have an annual incidence of 16–20 per million in Asian populations and 10–15 per million in Western countries, with a gradually rising global trend.^1,2^ Molecularly, GISTs are associated with mutations in receptor tyrosine kinase (RTK) genes such as *KIT* or *PDGFRA*.^3^ Although tyrosine kinase inhibitors like imatinib have significantly improved treatment outcomes, therapeutic resiance and postoperative recurrence remain major clinical challenges.^4^

Accurate recurrence risk assessment is critical for guiding postoperative strategies, and the modified NIH consensus criteria introduced in 2008 are commonly used.^5^ According to this framework, gastric GISTs smaller than 5 cm with a mitotic count of < 5 per 50 HPFs are typically classified as low risk. These cases generally carry a favourable prognosis and are often managed successfully with surgery alone. In contrast, tumors that exceed 5–10 cm in size, exhibit elevated mitotic counts (e.g., > 5 or > 10/50 HPFs), or are located in the non-gastric sites (such as the small bowel or colon) are considered intermediate or high-risk. Such features reflect enhanced biological aggressiveness, including increased potential for invasion and metastasis, thereby increasing the likelihood of postoperative recurrence. These patients often require prolonged adjuvant TKI therapy.^6^

Despite the diagnostic utility of immunohistochemical markers such as CD117 and DOG1, their ability to predict tumor behavior remains limited.^7^ This underscores the need for novel biomarkers to better characterize GIST aggressiveness. EphA3, a receptor tyrosine kinase in the Eph family, has been reported to be upregulated in various cancers and is associated with aggressive clinical features, including tumor invasion, metastasis, and poor prognosis in gastric and colorectal cancers.^8-11^ However, its expression and role in GIST have not yet been thoroughly examined.

This study aimed to investigate EPHA3 expression in GIST samples across different risk groups and evaluate its potential as a biomarker for risk stratification. We further explored the impact of EPHA3 on GIST cell proliferation, migration, and apoptosis, and investigated the underlying molecular mechanisms.

## Materials and methods

### Patient enrollment and tissue collection

Between 2023 and 2025, a total of 11 patients diagnosed with primary gastrointestinal stromal tumors (GIST) at The First Affiliated Hospital of Nankai University were recruited (Table 1). Eligible participants met the following criteria: (1) newly diagnosed and histologically confirmed GIST; (2) availability of representative tumor tissue blocks for immunohistochemical and molecular analyses; and (3) no prior exposure to neoadjuvant treatments such as radiotherapy, chemotherapy, or targeted therapy. Tumor specimens were excised during radical surgery and promptly frozen in liquid nitrogen to preserve RNA and protein integrity for downstream analyses. Following resection, clinical data and histopathological features were collected for each patient. The study was approved by the Ethics Committee of Nankai University (Approval No. NKUIRB2025078), and written informed consent was obtained from all subjects. All procedures were conducted in accordance with the Declaration of Helsinki.

**TABLE 1.** Clinicopathological characteristics of the study cohort (n = 11)

Case No.	Gender	Age	Primary site	Mitotic count (per 50 HPFs)	Ki-67 Index	NIH risk category	CD117 / DOG1	Maximum diameter (cm)*
1	Female	72	Stomach	10	5%	Intermediate	+/+	2
2	Female	61	Stomach	≤ 2	<5%	Intermediate	+/+	4
3	Male	39	Stomach	6	8%	Intermediate	+/+	3
4	Female	82	Stomach	< 5	5%	Intermediate	+/+	7
5	Male	51	Stomach	< 5	5%	Intermediate	+/+	3
6	Female	81	Stomach	> 20	15%	High	+/+	5
7	Female	68	Stomach	> 10	20%	High	+/+	3
8	Male	37	Stomach	> 20	10%	High	+/+	4.5
9	Male	61	Stomach	> 20	20%	High	+/+	7
10	Male	67	Rectum	> 10	15%	High	+/+	6.5
11	Female	57	Stomach	14	3%	High	+/-	3.5

HPF = high-power field; NIH = National Institutes of Health

* Tumor size was determined by the maximum diameter from the pathological gross description. Risk stratification was performed according to the modified NIH consensus criteria (2008).

### Histopathological evaluation and risk stratification

Histopathological evaluation was performed by two independent pathologists. The risk of recurrence for each GIST case was determined according to the modified National Institutes of Health (NIH) consensus criteria (2008), which integrates tumor size, primary site, and mitotic activity. Mitotic count was assessed by examining 50 consecutive high-power fields (HPFs, total area of 5 mm^2^) in the most cellular areas of the tumor. Tumors were specifically categorized into intermediate-risk and high-risk groups to compare the differential expression of EPHA3. For immunohistochemical (IHC) analysis, the scoring was conducted using a semi-quantitative system considering both staining intensity and the percentage of positive cells, as previously described. To ensure objectivity, the pathologists were blinded to the clinical risk stratification of the patients during the evaluation.

### Cell culture and transfection

GIST-430 cell line, derived from human gastrointestinal stromal tumor tissue, was generously provided by Professor Wenbing Ou from Zhejiang Sci-Tech University. Cells were maintained in DMEM (High glucose; SparkJade, CA0002), added with fetal bovine serum (Gibco, USA, 10%) and penicillin–streptomycin solution (MCE, HY-K1106, 1%). Cells were maintained at 37°C in a humidified atmosphere with 5% CO_2_.

To silence EPHA3 expression, GIST-430 cells were transfected with either EPHA3-specific siRNA (si-EPHA3) or a negative control (NC) siRNA. The siRNA sequences are provided in Supplementary Table S1. The small interfering RNA (siRNA) oligonucleotides were designed and manufactured by Saisofi Biotechnology Co., Ltd. Transient transfections were conducted using GP-transfect-mate reagent (GenePharma, G04008), following the recommended protocol, Cells were collected for further analysis 48 hours later. The efficacy of gene knockdown was subsequently examined with quantitative reverse transcription polymerase chain reaction (qRT-PCR) and Western blotting refer to Supplementary Table S2 (for detailed sequences).

### Establishment of EPHA3-knockout GIST-430 cells

To generate an EPHA3-deficient model, GIST-430 cells were genetically edited using a CRISPR/Cas9-based approach. A plasmid construct containing Cas9 nuclease and an sgRNA targeting human EPHA3 (Plasmid ID: L15525; supplier: Beyotime Biotechnology, China) was introduced into the cells using Lipofectamine™ 3000 transfection reagent (Thermo Fisher, L3000001), according to the manufacturer’s instructions. Approximately 48 hours after transfection, cells were subjected to puromycin selection at 2 μg/mL for 7–10 days to enrich stable knockout populations. EPHA3 disruption was subsequently validated by Western blotting, confirming the loss of protein expression in the established knockout cells.

### Immunohistochemistry (IHC)

Formalin-fixed, paraffin-embedded (FFPE) GIST specimens were sectioned at a thickness of 4 μm. The sections were deparaffinized in xylene and rehydrated through graded ethanol. Antigen retrieval was performed by microwave heating in 10 mM citrate buffer (pH 6.0) at medium power for approximately 10 minutes. Endogenous peroxidase activity was blocked with 3% hydrogen peroxide for 10 minutes at room temperature. After blocking non-specific binding, the sections were incubated overnight at 4°C with a primary monoclonal antibody against EPHA3 (Santa Cruz Biotechnology, sc-514209; dilution 1:1000). After three washes with PBS, an HRP-conjugated secondary antibody (ORIGENE, PV-8000-1) was applied for 1 hour at room temperature. Signal detection was performed using a DAB chromogen substrate, and nuclei were counterstained with hematoxylin. The slides were then dehydrated, mounted, and examined under a light microscope. EPHA3 expression was quantitatively assessed by average optical density (AOD) using ImageJ software.

### 5-ethynyl-2’-deoxyuridine (EdU) assay

To evaluate cell proliferation, 430-WT and 430-KO cells were seeded into 24-well plates and cultured under standard conditions until reaching appropriate confluence. Cell proliferation was assessed using the BeyoClick™ EdU Cell Proliferation Kit with Alexa Fluor 488 (Beyotime, C0071S) according to the manufacturer’s instructions. Briefly, cells were incubated with EdU working solution for an hour, then fixed with 4% paraformaldehyde and permeabilized with 0.3% Triton X-100. After washing with PBS, the cells were subjected to click reaction staining and counterstained with Hoechst 33342 to visualize nuclei. Images were captured using a fluorescence microscope, and the percentage of EdU-positive cells was quantified using ImageJ software. All experiments were performed in triplicate.

### Colony formation assay

For colony formation assays, a total of 300 cells with EPHA3 KO or WT were placed in 6-well plates and grown for 10 to 14 days. The colonies were then fixed using 4% paraformaldehyde and stained with a 0.1% crystal violet solution (Solarbio, G1064) for visualization, and quantified under a microscope.

### Apoptosis assay by flow cytometry

To determine apoptotic cell populations, exponentially growing GIST-430 cells were collected and rinsed twice with PBS. Using the Annexin V-FITC/PI staining kit (Yeasen, 40302ES60), apoptosis was detected as per the manufacturer’s protocols. Cells were treated with Annexin V-FITC and propidium iodide for 15 minutes at room temperature, keeping them in the dark to prevent photobleaching. Fluorescence signals were then acquired using a BD FACSCalibur flow cytometer. Data analysis and quantification of apoptotic fractions were performed using FlowJo software. All experimental groups were assessed in three independent replicates.

### Wound healing assay

In 6-well plates, GIST-430 cells were grown until they reached full coverage. A sterile pipette tip was used to make a scratch, and the cells were then cultured in a serum-free environment. Images were taken initially and after 24 h, and wound closure was quantified using ImageJ.

### Transwell assay

Cells were seeded in DMEM (2%FBS) into 8-μm pore Transwell inserts (Corning, 3422), with 10% FBS medium in the lower chamber. Cells that had migrated were fixed with 4% paraformaldehyde after 24 hours, stained with 0.1% crystal violet, and counted in five random areas.

### Western blot analysis

Total proteins were isolated using RIPA lysis buffer, and their concentrations were assessed with a BCA protein assay kit. Proteins in equal quantities were resolved on SDS-PAGE gels and then moved to 0.22 μm PVDF membranes provided by Millipore. To reduce nonspecific binding, membranes were incubated in 5% skim milk at room temperature for 1 hour. They were then incubated overnight at 4°C with the following primary antibodies: anti-EPHA3 (Santa Cruz, sc-514209, 1:1000), anti-PCNA (Santa Cruz, sc-71858, 1:1000), anti-Bcl-2 (CST, #3498, 1:1000), anti-Bax (CST, #2772, 1:1000), anti-cleaved Caspase-3 (ZenBio, L13OC01, 1:1000), anti-ZEB1 (CST, #70512T, 1:1000), anti-Vimentin (Proteintech, 10366-1-AP, 1:1000), and anti-N-cadherin (Proteintech, 22018-1-AP, 1:1000). After thorough washing, HRP-conjugated secondary antibodies (Proteintech, 1:1000) were applied for 60 minutes at ambient temperature. SparkJade ECL Super chemiluminescence reagent (ED0015) was used for detection, and ImageJ software counted the signal intensities of the protein bands.

### Quantitative real-time PCR (qRT-PCR)

The R1100 reagent from Solarbio was used to isolate total RNA. cDNA synthesis was performed using the EasyScript® All-in-One First-Strand cDNA Synthesis SuperMix for qPCR (TransGen, AE341), following the manufacturer’s protocol. Amplification was conducted on a Roche qPCR system utilizing PerfectStart® Green qPCR SuperMix (TransGen, AQ601). The thermal cycling program began with an initial denaturation at 94°C for 30 seconds, followed by 45 amplification cycles consisting of 94°C, 5 seconds and 60°C, 30 seconds. Gene expression was normalized and quantified using the 2^–ΔΔCt method. Supplementary Table S1 provides all the primer sequences utilized in the assay.

### RNA sequencing and bioinformatic analysis

Total RNA was obtained from 430-WT and EPHA3-knockout (430-KO) GIST-430 cells and submitted to Hongxu Bio (China) for transcriptome profiling. Library construction and high-throughput pairedend sequencing (150 bp, PE150) were performed on the Illumina NovaSeq platform. To ensure data quality, raw sequencing reads were processed with fastp^12^ for filtering and trimming. Clean reads were then aligned to the human reference genome (GRCh38) using HISAT2.^13^ Gene-level quantification was performed using featureCounts^14^, and differential gene expression analysis was conducted using DESeq2.^15^ Differentially expressed genes (DEGs) were filtered using the criteria of an adjusted P value < 0.05 and |log_2_ fold change| > 0.585, corresponding to a fold change > 1.5 for upregulated genes or < 0.67 for downregulated genes. Differentially expressed genes were visualized using a volcano plot, and the full list of DEGs was provided in the Supplementary Table S3. To investigate their functional implications, Gene Ontology (GO) and KEGG pathway enrichment analyses were performed using clusterProfiler^16^ and KEGG pathway annotation was based on the KEGG database.^17^

### Statistical analysis

Data are presented as mean ± SD from three independent experiments. Statistical analyses were performed using GraphPad Prism 9.0. Differences between two groups were assessed using unpaired two-tailed t-tests, and comparisons among multiple groups were analyzed by one-way ANOVA followed by Tukey’s post hoc test. Correlations between EPHA3 AOD values and tumor size or mitotic count grade were evaluated using Spearman correlation analysis. A *p* value < 0.05 was considered statistically significant. Significance levels are indicated as follows: *p* < 0.05 (*), *p* < 0.01 (**), *p* < 0.001 (***), and *p* < 0.0001 (****).

## Results

### EPHA3 positively correlates with malignant progression in GIST patients

**FIGURE 1.**
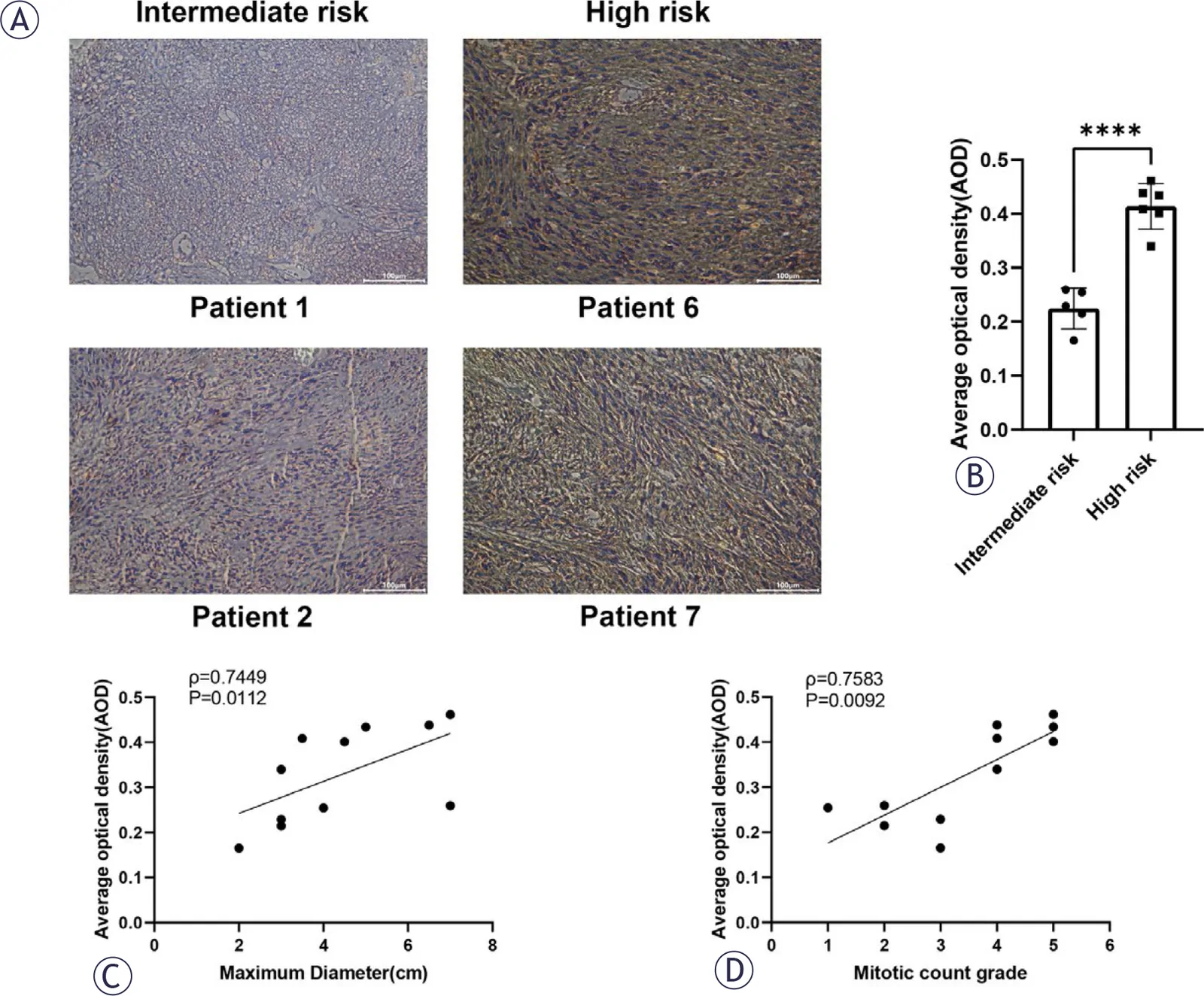
EPHA3 expression is elevated in high-risk GIST specimens and correlates with clinicopathological features. **(A)** Representative immunohistochemical (IHC) images of EPHA3 staining in intermediate-risk and high-risk GIST specimens, Scale bar = 100 μm. Full EPHA3 IHC images for all 11 patients are shown in Supplementary Figure S1, and the case numbers correspond to those listed in Table 1. **(B)** Quantification of EPHA3 staining by average optical density (AOD) in intermediaterisk and high-risk GIST specimens. Each dot represents one patient. Data are presented as the mean ± SD. ****p < 0.0001. **(C)** Spearman correlation analysis between maximum tumor diameter and EPHA3 AOD values in 11 GIST patients (ρ = 0.7449, *p* = 0.0112). **(D)** Spearman correlation analysis between mitotic count grade and EPHA3 AOD values in 11 GIST patients (ρ = 0.7583, *p* = 0.0092).

To assess the correlation between EPHA3 expression and GIST progression, immunohistochemistry was performed on tissue specimens from a pilot cohort of 11 patients, including six categorized as high-risk and five as intermediate-risk according to the modified NIH criteria. As shown in Figure 1A, representative IHC images revealed stronger EPHA3 staining in high-risk tumors than in intermediate-risk tumors. Quantitative analysis showed that EPHA3 AOD values were significantly elevated in high-risk tumors compared with intermediate-risk tumors (Figure 1B). Spearman correlation analysis further demonstrated that EPHA3 AOD values were positively correlated with tumor size (ϱ = 0.7449, p = 0.0112) and mitotic count grade (ϱ = 0.7583, p = 0.0092) (Figure 1C-D, Table 1). Full EPHA3 IHC images for all 11 patients are shown in Supplementary Figure 1. These results suggest that increased EPHA3 expression is associated with the high-risk clinical features of GIST, potentially reflecting its role in biological aggressiveness.

### EPHA3 promotes GIST tumor progression *in vitro*

**FIGURE 2.**
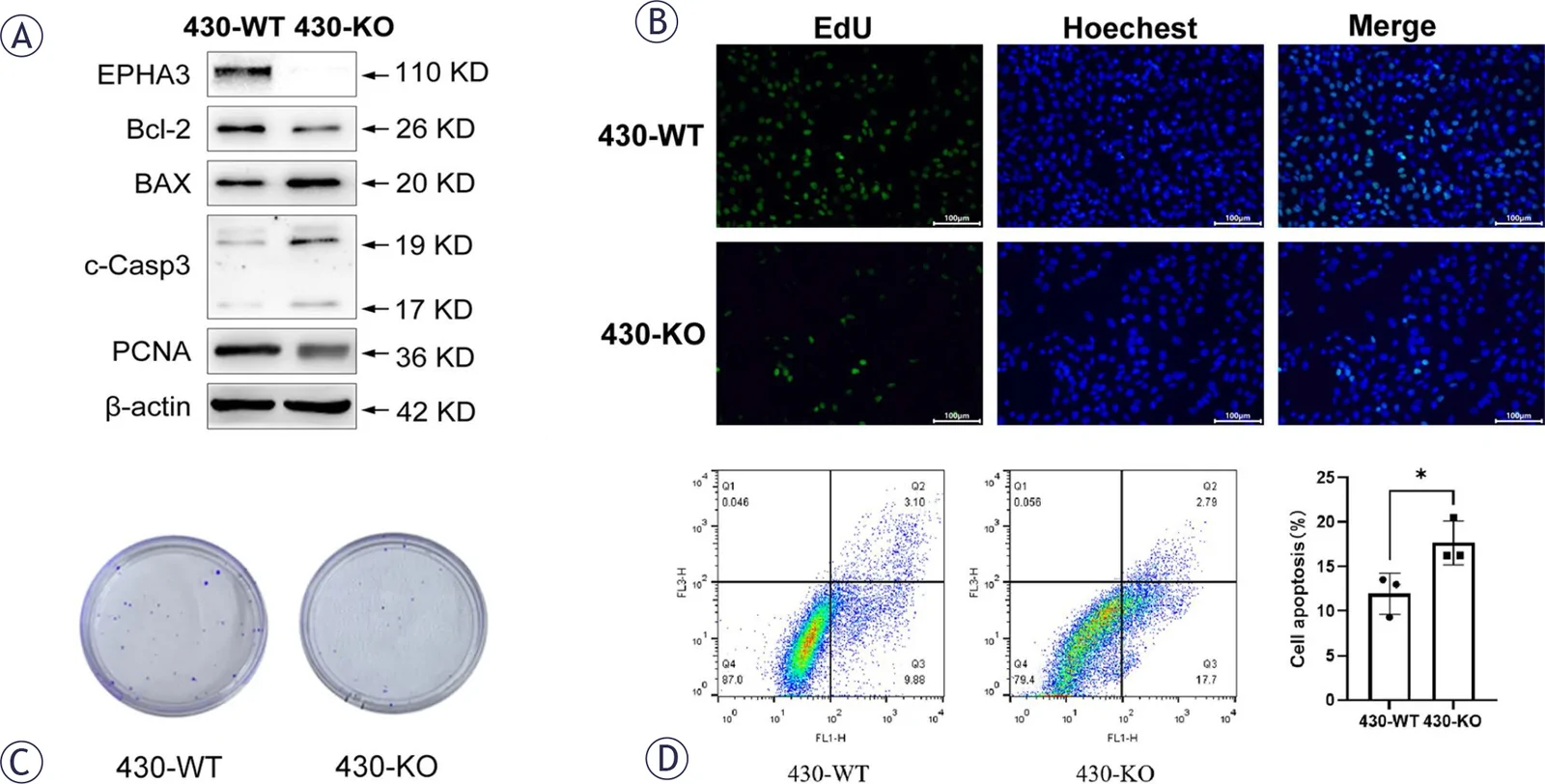
EPHA3 promotes the proliferation and survival of GIST-430 cells. **(A)** Representative Western blot images showing showing the expression of EPHA3, Bcl-2, Bax, cleaved caspase-3, and PCNA in 430-WT and 430-KO cells, with β-actin as the loading control. **(B)** 5-ethynyl-2’-deoxyuridine, EdU) assay showing the proliferative capacity of 430-WT and 430-KO cells, Scale bar = 100 μm. **(C)** Colony formation assay of 430-WT and 430-KO cells. **(D)** Representative flow cytometry plots and quantification of apoptotic cells in 430-WT and 430-KO cells using Annexin V/PI staining. Data are presented as mean ± SD from three independent experiments. *p < 0.05, **p < 0.01, ***p < 0.001.

To elucidate the functional contribution of EPHA3 in GIST, we employed a CRISPR/Cas9-mediated knockout strategy in GIST-430 cells. Representative Western blot images showed the loss of EPHA3 expression in 430-KO cells compared with 430-WT cells (Figure 2A, Supplementary Figure 2). Functional assays, including EdU and colony formation assays, demonstrated that EPHA3 depletion significantly attenuated cell proliferation (Figure 2B-C). In line with these observations, the expression of PCNA was notably decreased in 430-KO cells (Figure 2A, Supplementary Figure 2).

To assess whether EPHA3 loss triggers programmed cell death, we utilized Annexin V/PI double staining followed by flow cytometric analysis. A marked increase in apoptotic cell populations was observed in 430-KO cells relative to 430-WT cells (Figure 2D). Representative Western blot images further supported this finding, as EPHA3-deficient cells exhibited increased levels of cleaved caspase-3 and a reduced Bcl-2/Bax ratio (Figure 2A, Supplementary Figure 2). These data suggest that EPHA3 deficiency may activate the mitochondrial apoptosis pathway, thereby contributing to enhanced apoptotic activity and reduced tumor cell survival in GIST-430 cells.

### EPHA3 deficiency impairs migration and invasion of GIST-430 cells *in vitro*

To investigate the impact of EPHA3 in GIST cell mobility and invasive behavior, we conducted both wound healing and Transwell assays after silencing EPHA3 in GIST-430 cells.

**FIGURE 3.**
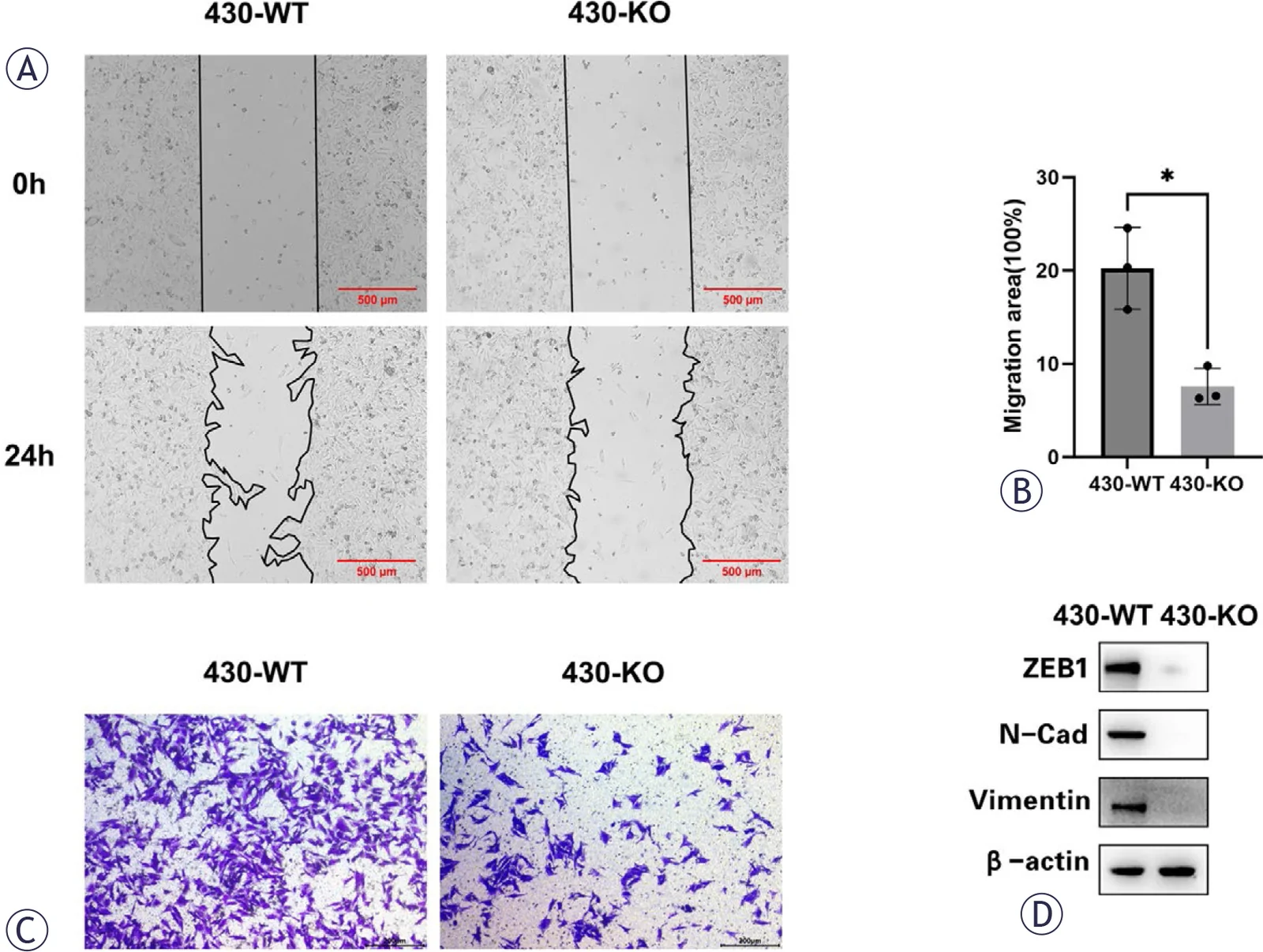
EPHA3 promotes the migration of GIST-430 cells. **(A)** Representative images of the wound-healing assay in 430-WT and 430-KO cells at 0 and 24 h. Scale bar = 500 μm. **(B)** Quantification of wound closure in 430-WT and 430-KO cells after 24 h. **(C)** Transwell migration assay showing the migratory ability of 430-WT and 430-KO cells. Scale bar = 200 μm. **(D)** Representative Western blot images showing ZEB1, N-cadherin, and Vimentin expression in 430-WT and 430-KO cells, with β-actin as the loading control. Data are presented as mean ± SD from three independent experiments. *p < 0.05, **p < 0.01, ***p < 0.001.

As anticipated, EPHA3 knockout significantly reduced the wound-closure rate of GIST-430 cells after 24 h, indicating impaired migratory capacity (Figure 3A–B). Consistently, the number of cells passing through the Transwell membrane was markedly decreased in 430-KO cells compared with 430-WT cells (Figure 3C). In line with these observations, representative Western blot images showed decreased expression of ZEB1, N-cadherin, and Vimentin in 430-KO cells (Figure 3D, Supplementary Figure 3). These results suggest that EPHA3 may regulate mesenchymal-related phenotypic remodeling and promote the migratory capacity of GIST-430 cells.

To control for potential off-target effects of EPHA3 depletion, we employed siRNA-mediated knockdown in GIST-430 cells. We also observed both a decreased migratory ability and down-regulated expression of relevant marker proteins in GIST-430 cells following EPHA3 knockdown (Supplementary Figure 4). Critically, both approaches yielded consistent phenotypic outcomes, confirming that the observed effects are specifically attributable to EPHA3 depletion rather than off-target or compensatory mechanisms.

### EPHA3 modulates the expression of genes associated with cell adhesion and matrix interactions in GIST cells

**FIGURE 4.**
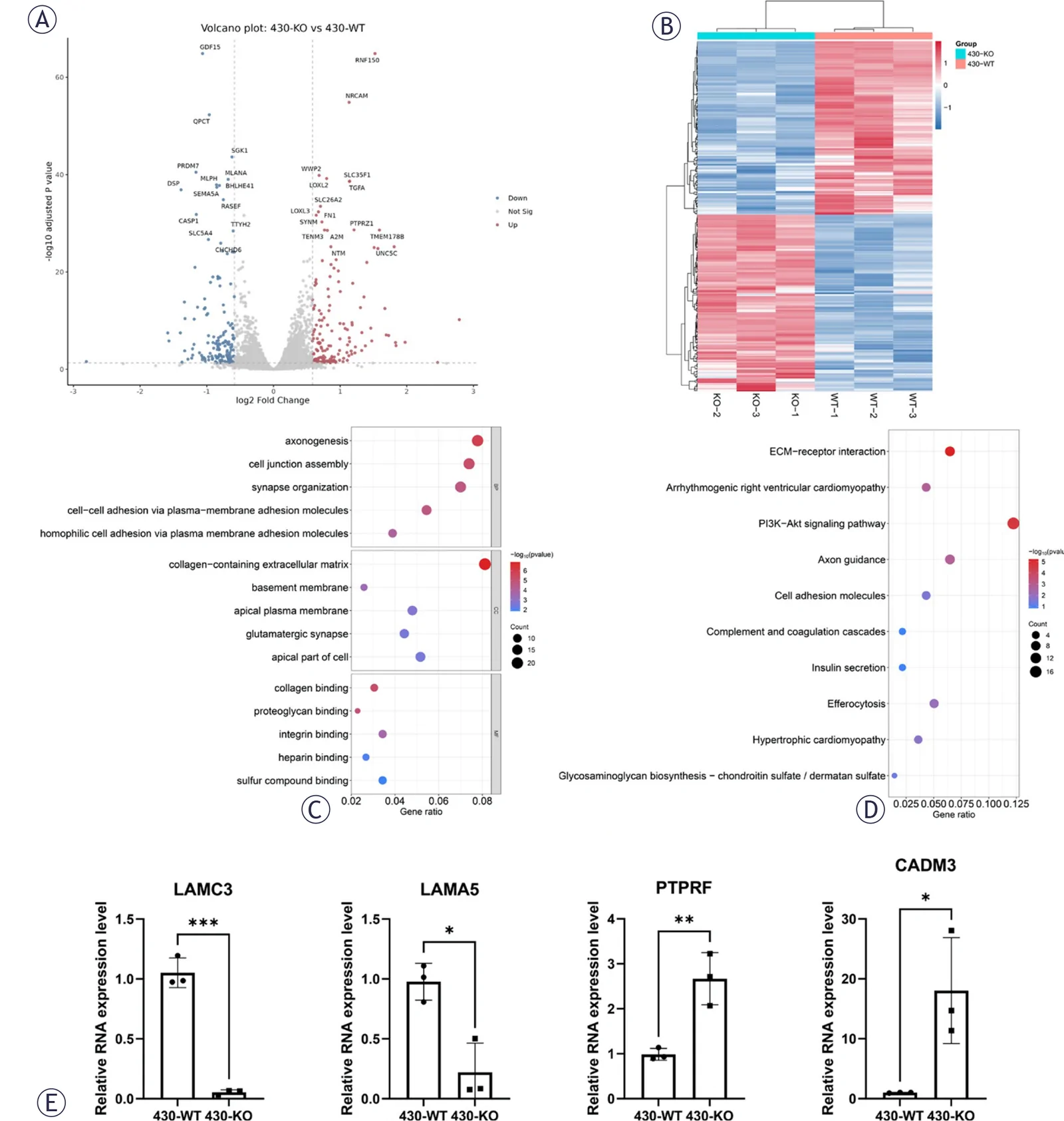
RNA sequencing (RNA-seq) analysis of EPHA3-knockout GIST-430 cells. **(A)** Volcano plot showing differentially expressed genes (DEGs) in 430-KO cells relative to 430-WT cells. DEGs were defined using the criteria of an adjusted P value < 0.05 and |log_2_ fold change| > 0.585. Red dots indicate upregulated genes, blue dots indicate downregulated genes, and grey dots indicate genes without significant differential expression. **(B)** Hierarchical-clustering heatmap showing the expression patterns of DEGs in 430-WT and 430-KO cells. Rows represent genes, columns represent samples, and the colour scale indicates row-wise Z-score-normalized expression levels. **(C)** Gene Ontology (GO) enrichment analysis showing the top five enriched terms in each GO category, including biological process, cellular component, and molecular function. **(D)** Kyoto Encyclopaedia of Genes and Genomes (KEGG) pathway-enrichment analysis showing the top 10 enriched pathways. (E) RT-qPCR validation of the relative mRNA expression levels of LAMC3, LAMA5, PTPRF, and CADM3 in 430-WT and 430-KO cells. Data are presented as the mean ± SD from three independent experiments. *p < 0.05, **p < 0.01, ***p < 0.001.

To elucidate the potential role of EPHA3 in the progression of gastrointestinal stromal tumors (GIST), we conducted a comparative transcriptomic analysis using bulk RNA sequencing in 430-WT cells and their EPHA3-knockout (430-KO) counterparts. Differential expression analysis identified a total of 327 significantly altered genes, including 162 upregulated and 165 downregulated genes, as visualized in the volcano plot (Figure 4A). Hierarchical clustering analysis further revealed distinct gene-expression patterns between 430-WT and 430-KO cells (Figure 4B). The full list of differentially expressed genes is provided in Supplementary Table S2. Functional enrichment through Gene Ontology (GO) analysis indicated that these differentially expressed genes were predominantly involved in biological processes related to cell-cell adhesion, homophilic cell adhesion, and cell junction assembly. Regarding cellular components, enrichment was observed in collagen-containing extracellular matrix and apical plasma membrane. Molecular function categories significantly represented included proteoglycan binding, integrin binding, and collagen binding (Figure 4C). Additionally, KEGG pathway analysis highlighted the enrichment of several pathways associated with tumor invasiveness and mesenchymal remodeling, including extracellular matrix (ECM)-receptor interactions, PI3K-Akt signaling, and cell adhesion (Figure 4D).^18^ Among the identified differentially expressed genes, key mediators of cell-matrix interactions and adhesion, including LAMC3, LAMA5, PTPRF and CADM3 were selected, and their altered expression levels were confirmed by RT-qPCR (Figure 4E). Collectively, these data suggest that EPHA3 may regulate signaling pathways associated with cell adhesion, extracellular matrix interaction, and mesenchymal-related phenotypic remodeling in GIST cells.

## Discussion

The biological role of EPHA3 in determining phenotypic characteristics across histopathological subtypes of GIST, particularly in high- and intermediate-risk groups, remains poorly understood. In the present study, we found that EPHA3 expression was elevated in high-risk GIST specimens and was associated with larger tumor size and higher mitotic activity. In functional experiments using GIST-430 cells, EPHA3 deficiency significantly suppressed cell proliferation and migration while promoting apoptosis. Furthermore, transcriptomic profiling suggested that EPHA3 may contribute to the malignant phenotype of GIST cells through the regulation of pathways related to cell adhesion, extracellular matrix interaction, and mesenchymal-related phenotypic remodeling. Nevertheless, several limitations should be acknowledged. The clinical cohort in this study was relatively small, and the *in vitro* functional analyses were performed mainly in a single GIST-430 cell model. In addition, although our findings support an association between EPHA3 expression and aggressive clinicopathological features, the prognostic value of EPHA3 still requires validation in larger independent cohorts.

Eph receptors and their cognate ephrin ligands play pivotal roles in diverse biological processes, including cell adhesion, migration^19^, and angiogenesis.^20^ Although their expression in normal adult tissues is typically restricted, Eph/ephrin family members are frequently overexpressed in human malignancies^21^, where they are often associated with aggressive, invasive, and metastatic behavior.^22-24^ As cell surface receptors, Eph/ephrin signaling can elicit distinct biological outcomes, ranging from cell adhesion to repulsion, depending on multiple contextual factors. Emerging evidence suggests that these opposing effects are modulated by the intensity of Eph receptor activation, the specific cellular context, and receptor density at the cell surface.^25,26^ Crucially, EPHA3 acts as a key signaling hub, transmitting extracellular cues into intracellular responses that drive oncogenic potential across multiple malignancies. In glioblastoma multiforme (GBM), EPHA3 demonstrates minimal expression in normal brain tissue but is markedly upregulated in glioblastoma stem cells (GSCs), where it critically maintains self-renewal capacity and cell survival. Notably, EPHA3 knockdown induces tumor cell differentiation and apoptosis in GBM models.^27^

The Eph receptor family, including EPHA2, EPHA3, EPHB2, and EPHB4, is frequently dysregulated in breast cancer. Comparative analyses reveal significantly elevated expression of these receptors in malignant versus normal mammary epithelium. Specifically, EPHB2 overexpression occurs in 51% of breast tumors and correlates with unfavorable clinical outcomes. EPHB4 expression similarly associates with advanced tumor stage, higher histological grade, increased proliferation indices, and DNA aneuploidy.^28^ Functional studies demonstrate that EPHB4 knockdown reduces cell viability, enhances apoptosis, and sensitizes breast cancer cells to TRAIL-induced cell death.^29^ EPHA2 overexpression also portends worse prognosis in breast cancer, where it potentiates HER2-mediated oncogenic signaling cascades. Genetic ablation of EPHA2 in murine models significantly attenuates mammary tumor progression and metastasis.^30^ In pancreatic cancer, EPHA2 knockdown has been reported to reduce transwell invasion in specific molecular subtypes.^31^ Furthermore, EPHA3 has been identified as a novel effector of RAGE-mediated motility in breast cancer cells.^24^ Moreover, higher levels of EPHA4, EPHA7, and EPHA10 expression are linked to adverse clinical outcomes in breast cancer, with EPHA4 specifically promoting tumor progression through TGFβ pathway activation. These collective findings underscore the pivotal role of Eph receptors in driving oncogenic processes across diverse malignancies by orchestrating complex intracellular signaling networks.

## Conclusions

In conclusion, our study demonstrates that EPHA3 expression is remarkably elevated in high-risk GISTs compared to intermediate-risk cases, with its overexpression significantly correlating with aggressive features. Functional studies revealed that EPHA3 knockdown limits tumor cell proliferation, invasion, and migration by modulating pathways associated with mesenchymal phenotypic remodeling and cell adhesion. suggest that EPHA3 may serve as a promising novel therapeutic target and a potential candidate biomarker for the risk assessment of GIST.

## Supplementary Material

Supplementary Material Details

## References

[1] Sircar K, Hewlett BR, Huizinga JD, Chorneyko K, Berezin I, Riddell RH. (1999). Interstitial cells of cajal as precursors of gastrointestinal stromal tumors. Am J Surg Pathol.

[2] Xu Y, Peng XL, East MP, Mccabe IC, Stroman GC, Jenner MR (2025). Tumorintrinsic kinome landscape of pancreatic cancer reveals new therapeutic approaches. Cancer Discov.

[3] Venkataraman V, George S, Cote GM. (2023). Molecular advances in the treatment of advanced gastrointestinal stromal tumor. Oncologist.

[4] Buchdunger E, Cioffi CL, Law N, Stover D, Ohno-Jones S, Druker BJ (2000). Abl protein-tyrosine kinase inhibitor STI571 inhibits in vitro signal transduction mediated by c-kit and platelet-derived growth factor receptors. J Pharmacol Exp Ther.

[5] Joensuu H. (2008). Risk stratification of patients diagnosed with gastrointestinal stromal tumor. Hum Pathol.

[6] Joensuu H, Eriksson M, Sundby Hall K, Hartmann JT, Pink D, Schutte J (2012). One vs three years of adjuvant imatinib for operable gastrointestinal stromal tumor: a randomized trial. JAMA.

[7] Novelli M, Rossi S, Rodriguez-Justo M, Taniere P, Seddon B, Toffolatti L (2010). DOG1 and CD117 are the antibodies of choice in the diagnosis of gastrointestinal stromal tumours. Histopathology.

[8] Xi H, Wu X, Wei B, Chen L. (2012). Aberrant expression of EphA3 in gastric carcinoma: correlation with tumor angiogenesis and survival. J Gastroenterol.

[9] Li M, Yang C, Liu X, Yuan L, Zhang F, Wang M (2016). EphA3 promotes malignant transformation of colorectal epithelial cells by upregulating oncogenic pathways. Cancer Lett.

[10] Nasri B, Inokuchi M, Ishikawa T, Uetake H, Takagi Y, Otsuki S (2017). High expression of EphA3 (erythropoietin-producing hepatocellular a3) in gastric cancer is associated with metastasis and poor survival. BMC Clin Pathol.

[11] Surawska H, Ma PC, Salgia R. (2004). The role of ephrins and eph receptors in cancer. Cytokine Growth Factor Rev.

[12] Chen S, Zhou Y, Chen Y, Gu J. (2018). Fastp: an ultra-fast all-in-one FASTQ preprocessor. Bioinformatics.

[13] Kim D, Paggi JM, Park C, Bennett C, Salzberg SL. (2019). Graph-based genome alignment and genotyping with HISAT2 and HISAT-genotype. Nat Biotechnol.

[14] Liao Y, Smyth GK, Shi W. (2014). Featurecounts: an efficient general purpose program for assigning sequence reads to genomic features. Bioinformatics.

[15] Love MI, Huber W, Anders S. (2014). Moderated estimation of fold change and dispersion for RNA-seq data with DESeq2. Genome Biol.

[16] Wu T, Hu E, Xu S, Chen M, Guo P, Dai Z (2021). Clusterprofiler 4.0: a universal enrichment tool for interpreting omics data. Innovation (N Y).

[17] Kanehisa M, Furumichi M, Sato Y, Matsuura Y, Ishiguro-Watanabe M. (2025). KEGG: biological systems database as a model of the real world. Nucleic Acids Res.

[18] Kanehisa M, Goto S. (2000). KEGG: kyoto encyclopedia of genes and genomes. Nucleic Acids Res.

[19] Park I, Lee H. (2015). EphB/ephrinb signaling in cell adhesion and migration. Mol Cells.

[20] Adams RH, Wilkinson GA, Weiss C, Diella F, Gale NW, Deutsch U (1999). Roles of ephrinb ligands and EphB receptors in cardiovascular development: demarcation of arterial/venous domains, vascular morphogenesis, and sprouting angiogenesis. Genes Dev.

[21] Anderton M, van der Meulen E, Blumenthal MJ, Schafer G. (2021). The role of the eph receptor family in tumorigenesis. Cancers (Basel).

[22] Chen J. (2012). Regulation of tumor initiation and metastatic progression by Eph receptor tyrosine kinases. Adv Cancer Res.

[23] Kandouz M. (2012). The eph/ephrin family in cancer metastasis: communication at the service of invasion. Cancer Metastasis Rev.

[24] Talia M, Cirillo F, Spinelli A, Zicarelli A, Scordamaglia D, Muglia L (2023). The ephrin tyrosine kinase A3 (EphA3) is a novel mediator of RAGE-prompted motility of breast cancer cells. J Exp Clin Cancer Res.

[25] Pasquale EB. (2010). Eph receptors and ephrins in cancer: bidirectional signalling and beyond. Nat Rev Cancer.

[26] Nievergall E, Lackmann M, Janes PW. (2012). Eph-dependent cell-cell adhesion and segregation in development and cancer. Cell Mol Life Sci.

[27] Offenhauser C, Al-Ejeh F, Puttick S, Ensbey KS, Bruce ZC, Jamieson PR (2018). EphA3 pay-loaded antibody therapeutics for the treatment of glioblastoma. Cancers (Basel).

[28] Wu Q, Suo Z, Risberg B, Karlsson MG, Villman K, Nesland JM. (2004). Expression of ephb2 and ephb4 in breast carcinoma. Pathol Oncol Res.

[29] Kumar SR, Singh J, Xia G, Krasnoperov V, Hassanieh L, Ley EJ (2006). Receptor tyrosine kinase EphB4 is a survival factor in breast cancer. Am J Pathol.

[30] Brantley-Sieders DM, Zhuang G, Hicks D, Fang WB, Hwang Y, Cates JMM (2008). The receptor tyrosine kinase EphA2 promotes mammary adenocarcinoma tumorigenesis and metastatic progression in mice by amplifying ErbB2 signaling. J Clin Invest.

[31] Duxbury MS, Ito H, Zinner MJ, Ashley SW, Whang EE. (2004). Retraction note: EphA2: a determinant of malignant cellular behavior and a potential therapeutic target in pancreatic adenocarcinoma. Oncogene.

