# Supplementary material for: EPHA3, upregulated in high-risk histological subtypes of gastrointestinal stromal tumor, promotes GIST progression
Source: Radiol Oncol. 2026 Sep 7;60(3):416–26. doi: 10.2478/raon-2026-0042 (PMC13548678; doi:10.2478/raon-2026-0042)
Supplement: Supplementary file 1 [file raon-2026-0042_sm.pdf]

EPHA3, upregulated in high-risk histological subtypes of gastrointestinal stromal tumor, promotes GIST progression

Han Wei, Jia Zhao, Hongmei Wang, Lihong Guo, Yanhong Liu, Chuntao Wang, Liang Yang, Zhi Qi, Wencong Tian, Lei Cao, Yang Gao

doi: 10.2478/raon-2026-0042

Radiol Oncol 2026; 60(3): 416-426.

Supplementary tables

SUPPLEMENTARY TABLE S1. siRNA sequences used for transfection

Target gene (Species)	Sequence (5'→3')
si-EPHA3 (*Homo sapiens*)	Forward: CCAGGUUUCUACAAGGCAUTT<br>Reverse: AUGCCUUGUAGAAACCUGGTT
si-NC	Forward: UUCUCCGAACGUGUCACGUTT<br>Reverse: ACGUGACACGUUCGGAGAATT

SUPPLEMENTARY TABLE S2. Primer sequences for quantitative reverse transcription polymerase chain reaction (qRT-PCR)

Gene (Species)	Sequence (5'→3')
EPHA3 (*Homo sapiens*)	Forward: CAGCCTTCCAACGAAGTTAATCT<br>Reverse: TGCACACCTGGTAAGTCCTGA
ZEB1 (*Homo sapiens*)	Forward: GATGATGAATGCGAGTCAGATGC<br>Reverse: ACAGCAGTGTCTTGTTGTTGT
VIM (*Homo sapiens*)	Forward: GACGCCATCAACACCGAGTT<br>Reverse: CTTTGTCGTTGGTTAGCTGGT
CDH1 (*Homo sapiens*)	Forward: ATTTTTCCCTCGACACCCGAT<br>Reverse: TCCCAGGCGTAGACCAAGA
CDH2 (*Homo sapiens*)	Forward: TGCGGTACAGTGTAACTGGG<br>Reverse: GAAACCGGGCTATCTGCTCG
NRCAM (*Homo sapiens*)	Forward: TCCAACCATCACCCAACAGTC<br>Reverse: TGAGTCCCATTACGGGTCCAG
LAMC3 (*Homo sapiens*)	Forward: CCCACCTCGGTCAACATCAC<br>Reverse: GAGGCGCTGTAGAACTGGTA
LAMA5 (*Homo sapiens*)	Forward: GACTGCCAACAGTGCCAAC<br>Reverse: CCACCCTGATAGGTGCCAT
PTPRF (*Homo sapiens*)	Forward: ACAGGACGGGTTGGCTACA<br>Reverse: AAGTTCAGCTCCTTCGTCAGA
CADM3 (*Homo sapiens*)	Forward: CAAGTGCCAAGTGAAAGATCACG<br>Reverse: CCCAAAGTAGAGAGTCTGCTGAG
ACTB (*Homo sapiens*)	Forward: CATGTACGTTGCTATCCAGGC<br>Reverse: CTCCTTAATGTCACGCACGAT

SUPPLEMENTARY TABLE S3. List of differentially expressed genes

Geneid	gene_name	gene_type	mean_norm_430	mean_norm_430_KO	log_2FoldChange	pvalue	padj	change
ENSG00000002586.20	CD99	protein_coding	345.2539762	897.5698055	1.378337928	5.93E-07	1.54E-05	Up
ENSG00000004799.8	PDK4	protein_coding	151.2838665	93.4654943	-0.693056402	0.00022553	0.002365029	Down
ENSG00000005471.19	ABCB4	protein_coding	158.9134479	94.42341444	-0.750226418	0.000228784	0.002388864	Down
ENSG00000023171.20	GRAMD1B	protein_coding	594.973511	371.8604592	-0.677148855	4.01E-12	2.98E-10	Down
ENSG00000030419.17	IKZF2	protein_coding	79.84019267	146.5099774	0.875303268	0.000223591	0.002353664	Up
ENSG00000041515.16	MYO16	protein_coding	127.0564942	79.60404924	-0.671982336	0.000905216	0.007165779	Down
ENSG00000047365.13	ARAP2	protein_coding	164.4379476	249.9371611	0.605474279	5.16E-05	0.000703593	Up
ENSG00000049192.15	ADAMTS6	protein_coding	40.77079228	70.86220976	0.799335513	0.003159224	0.0187421	Up
ENSG00000054793.14	ATP9A	protein_coding	93.6003788	54.95121476	-0.772079879	0.001856632	0.01249308	Down
ENSG00000055163.21	CYFIP2	protein_coding	340.3044991	214.8554005	-0.663731289	2.84E-07	7.99E-06	Down
ENSG00000057294.18	PKP2	protein_coding	145.0050529	92.71302335	-0.644099492	0.000598259	0.005090754	Down
ENSG00000064300.9	NGFR	protein_coding	560.4386346	988.7401142	0.81857115	1.79E-19	3.15E-17	Up
ENSG00000067715.15	SYT1	protein_coding	50.37120931	94.76195874	0.912666458	0.000301316	0.002967377	Up
ENSG00000071073.13	MGAT4A	protein_coding	166.9510832	95.11455667	-0.810807354	4.10E-06	8.22E-05	Down
ENSG00000071991.9	CDH19	protein_coding	699.6697453	1311.585038	0.907462748	6.20E-24	1.66E-21	Up
ENSG00000074047.22	GLI2	protein_coding	95.08232789	189.7327734	0.997064602	1.47E-08	5.66E-07	Up
ENSG00000076356.7	PLXNA2	protein_coding	284.0656858	124.2508905	-1.189436571	5.83E-13	5.02E-11	Down
ENSG00000077238.14	IL4R	protein_coding	137.7799021	79.65286269	-0.792491567	0.000123693	0.001452172	Down
ENSG00000080573.7	COL5A3	protein_coding	432.6367254	650.8325668	0.590052271	1.72E-09	8.00E-08	Up
ENSG00000080709.17	KCNN2	protein_coding	299.6257562	181.7546209	-0.720965612	4.08E-08	1.44E-06	Down
ENSG00000081923.15	ATP8B1	protein_coding	29.99664842	59.62237	0.991969056	0.002135736	0.013989671	Up
ENSG00000082293.13	COL19A1	protein_coding	1506.276312	2288.804128	0.603418882	2.59E-15	2.92E-13	Up
ENSG00000082482.14	KCNK2	protein_coding	50.26947495	84.20166668	0.746369885	0.006984219	0.03452292	Up
ENSG00000082497.12	SERTAD4	protein_coding	151.7832349	401.0302754	1.400623023	3.84E-25	1.12E-22	Up
ENSG00000084636.18	COL16A1	protein_coding	206.7272171	313.9629608	0.60305668	4.22E-05	0.000595958	Up
ENSG00000085276.19	MECOM	protein_coding	156.3068898	445.5058582	1.510192863	2.46E-28	9.87E-26	Up
ENSG00000088038.20	CNOT3	protein_coding	124.2818395	197.7414983	0.668506807	0.000741475	0.006087397	Up
ENSG00000091129.22	NRCAM	protein_coding	3450.481201	7573.101337	1.134150268	3.30E-59	1.41E-55	Up
ENSG00000096696.16	DSP	protein_coding	805.9803697	308.1991581	-1.386316066	1.57E-40	1.34E-37	Down
ENSG00000099139.14	PCSK5	protein_coding	448.5840845	683.2145939	0.607062983	1.38E-10	7.77E-09	Up
ENSG00000100167.21	SEPTIN3	protein_coding	204.255094	129.1565044	-0.658616747	0.000304311	0.002987692	Down
ENSG00000100191.6	SLC5A4	protein_coding	992.3045367	504.0355322	-0.976539367	4.70E-30	2.15E-27	Down
ENSG00000100628.12	ASB2	protein_coding	193.3631215	120.7757116	-0.679606292	0.000685132	0.005694051	Down
ENSG00000101197.13	BIRC7	protein_coding	858.4654403	484.215673	-0.826259404	5.46E-22	1.25E-19	Down
ENSG00000101210.14	EEF1A2	protein_coding	1018.184662	528.8827452	-0.944819815	6.90E-21	1.38E-18	Down
ENSG00000101230.6	ISM1	protein_coding	176.2358491	89.0110982	-0.983713642	1.21E-07	3.77E-06	Down
ENSG00000101265.16	RASSF2	protein_coding	195.6718889	124.2938598	-0.65498685	9.89E-05	0.001205188	Down
ENSG00000101850.13	GPR143	protein_coding	1499.454346	882.4378926	-0.764977349	1.33E-27	5.03E-25	Down
ENSG00000102109.9	PCSK1N	protein_coding	107.0294298	58.49366358	-0.868161805	0.000160363	0.001801905	Down
ENSG00000102313.9	ITIH6	protein_coding	335.9305625	567.9337057	0.758263188	3.00E-13	2.69E-11	Up
ENSG00000103489.12	XYLT1	protein_coding	2720.071205	4235.387013	0.639217838	1.76E-21	3.82E-19	Up
ENSG00000105088.9	OLFM2	protein_coding	242.3104159	138.8541596	-0.801653991	2.65E-07	7.56E-06	Down
ENSG00000105613.10	MAST1	protein_coding	220.104621	144.0801223	-0.613029287	0.000241147	0.002489455	Down
ENSG00000105618.14	PRPF31	protein_coding	27.8719355	153.556756	2.462135765	0.008626885	0.040467676	Up
ENSG00000105708.9	ZNF14	protein_coding	111.9392512	187.1395145	0.740385923	9.43E-06	0.000166182	Up
ENSG00000105711.14	SCN1B	protein_coding	138.9898782	89.34720501	-0.639478992	0.001489553	0.010519509	Down
ENSG00000106069.26	CHN2	protein_coding	716.0573747	472.6525501	-0.597812976	1.62E-08	6.18E-07	Down
ENSG00000106278.12	PTPRZ1	protein_coding	313.3382477	723.676303	1.208071677	4.14E-32	2.31E-29	Up
ENSG00000107281.10	NPDC1	protein_coding	460.22346	285.7009522	-0.685852155	2.16E-07	6.32E-06	Down
ENSG00000107719.10	PALD1	protein_coding	150.1351944	95.25077388	-0.656200989	0.000743662	0.006097555	Down
ENSG00000109738.11	GLRB	protein_coding	28.99428826	52.23735448	0.85133826	0.007911843	0.037981581	Up
ENSG00000111341.10	MGP	protein_coding	38.10001377	85.61702721	1.173155423	5.51E-05	0.000746468	Up
ENSG00000112280.18	COL9A1	protein_coding	104.7877492	268.3732006	1.357138089	9.36E-18	1.40E-15	Up
ENSG00000112294.14	ALDH5A1	protein_coding	88.79707708	38.25024531	-1.214170181	3.09E-06	6.44E-05	Down
ENSG00000112902.12	SEMA5A	protein_coding	4065.499859	2254.861432	-0.850319789	4.40E-41	4.03E-38	Down
ENSG00000113369.9	ARRDC3	protein_coding	838.0071419	1270.326493	0.599762858	4.15E-15	4.51E-13	Up
ENSG00000113594.10	LIFR	protein_coding	365.565615	599.4975304	0.712400692	2.30E-11	1.46E-09	Up
ENSG00000114019.15	AMOTL2	protein_coding	613.0770972	958.032944	0.643382927	1.21E-11	8.14E-10	Up
ENSG00000114251.15	WNT5A	protein_coding	352.0004434	527.6321113	0.585008342	4.58E-08	1.58E-06	Up
ENSG00000115232.14	ITGA4	protein_coding	268.6158039	434.8924468	0.693964918	1.17E-09	5.58E-08	Up
ENSG00000115318.12	LOXL3	protein_coding	3771.839004	6022.416076	0.675058787	6.23E-36	4.44E-33	Up
ENSG00000115339.14	GALNT3	protein_coding	349.8591183	230.8783606	-0.600341726	5.76E-06	0.000109249	Down
ENSG00000115414.21	FN1	protein_coding	8631.587316	14281.10973	0.726384987	9.30E-34	5.43E-31	Up
ENSG00000115457.10	IGFBP2	protein_coding	646.0247472	422.7256339	-0.610460165	2.19E-09	9.92E-08	Down
ENSG00000115525.18	ST3GAL5	protein_coding	2252.823029	1393.830285	-0.692641264	5.22E-27	1.81E-24	Down
ENSG00000115648.14	MLPH	protein_coding	2039.201106	1128.030775	-0.854044712	1.15E-41	1.23E-38	Down
ENSG00000115828.17	QPCT	protein_coding	7107.62132	3645.223779	-0.963189208	1.58E-56	5.07E-53	Down
ENSG00000116962.15	NID1	protein_coding	99.17562033	244.9039663	1.305538543	1.92E-15	2.20E-13	Up
ENSG00000117983.17	MUC5B	protein_coding	145.2867051	230.1151943	0.663968705	9.55E-06	0.000167851	Up
ENSG00000118503.18	TNFAIP3	protein_coding	853.7247352	489.6278016	-0.802949736	4.06E-20	7.55E-18	Down
ENSG00000118513.21	MYB	protein_coding	241.6374247	137.1026923	-0.817220605	2.06E-07	6.06E-06	Down
ENSG00000118515.12	SGK1	protein_coding	9617.993528	6252.255164	-0.621275251	9.16E-48	2.35E-44	Down
ENSG00000118946.12	PCDH17	protein_coding	27.89699024	92.41494077	1.728134725	2.53E-09	1.13E-07	Up
ENSG00000120215.10	MLANA	protein_coding	11244.28191	7020.962495	-0.679347005	6.72E-43	9.58E-40	Down
ENSG00000122863.6	CHST3	protein_coding	215.4555838	491.6463561	1.188352339	1.10E-20	2.18E-18	Up
ENSG00000123095.6	BHLHE41	protein_coding	2748.956645	1568.435366	-0.809523588	1.74E-41	1.71E-38	Down
ENSG00000124772.12	CPNE5	protein_coding	67.7274952	26.92846756	-1.331966083	9.13E-06	0.000161569	Down
ENSG00000125378.16	BMP4	protein_coding	85.92459023	53.34410402	-0.688676563	0.010926139	0.048180144	Down
ENSG00000125726.11	CD70	protein_coding	393.3215464	253.7018595	-0.633329418	5.96E-06	0.000112624	Down
ENSG00000125848.10	FLRT3	protein_coding	40.89166797	87.88911132	1.101676551	2.40E-05	0.000369757	Up
ENSG00000125864.14	BFSP1	protein_coding	78.20285099	123.158007	0.658329849	0.003845391	0.021868317	Up
ENSG00000126856.16	PRDM7	protein_coding	1057.772344	472.5632057	-1.162052183	1.53E-44	3.27E-41	Down
ENSG00000127220.6	ABHD8	protein_coding	221.8383879	147.0169643	-0.593972078	0.000175611	0.00193595	Down
ENSG00000127952.17	STYXL1	protein_coding	119.5582423	74.94966971	-0.67304327	0.001623961	0.011313063	Down
ENSG00000128266.9	GNAZ	protein_coding	93.39047867	140.7376322	0.592749303	0.003278744	0.019281781	Up
ENSG00000128805.15	ARHGAP22	protein_coding	221.9378603	137.2951993	-0.69326428	7.86E-06	0.000141864	Down
ENSG00000129595.14	EPB41L4A	protein_coding	93.22856835	142.6159236	0.615625102	0.00142812	0.010192235	Up
ENSG00000130203.10	APOE	protein_coding	362.4674651	184.5338796	-0.97373918	2.77E-09	1.23E-07	Down
ENSG00000130303.14	BST2	protein_coding	75.66012616	44.61750188	-0.759793804	0.005784191	0.029838853	Down
ENSG00000130513.7	GDF15	protein_coding	3856.741082	1846.170165	-1.063456088	2.17E-69	1.39E-65	Down
ENSG00000130702.15	LAMA5	protein_coding	693.4763909	454.7570251	-0.607753259	7.07E-11	4.14E-09	Down
ENSG00000132182.13	NUP210	protein_coding	293.7963041	133.5787937	-1.13864711	1.51E-15	1.75E-13	Down
ENSG00000134013.16	LOXL2	protein_coding	1755.355158	3051.762443	0.797462047	4.03E-43	6.47E-40	Up

Geneid	gene_name	gene_type	mean_norm_430	mean_norm_430_KO	log_2FoldChange	pvalue	padj	change
ENSG00000134986.14	NREP	protein_coding	71.0721422	117.6557072	0.727437345	0.00055215	0.004797017	Up
ENSG00000135116.9	HRK	protein_coding	313.5374269	192.3254176	-0.704339531	7.42E-08	2.43E-06	Down
ENSG00000135119.15	RNFT2	protein_coding	132.6629547	75.49262178	-0.815364411	0.000146413	0.001668533	Down
ENSG00000135127.12	BICDL1	protein_coding	334.2693279	198.1962404	-0.753617906	1.91E-09	8.79E-08	Down
ENSG00000135362.14	PRR5L	protein_coding	79.83790851	40.67186719	-0.973438646	0.000265188	0.002696428	Down
ENSG00000135424.19	ITGA7	protein_coding	234.8246231	125.1215492	-0.906906587	1.01E-07	3.21E-06	Down
ENSG00000136040.9	PLXNC1	protein_coding	1616.936731	925.5742077	-0.804150621	8.15E-22	1.84E-19	Down
ENSG00000137337.16	MDC1	protein_coding	64.38991769	117.8195378	0.870768773	0.002628302	0.016320369	Up
ENSG00000137460.10	FHDC1	protein_coding	264.0348614	416.9802638	0.658176806	1.29E-08	5.03E-07	Up
ENSG00000137571.11	SLCO5A1	protein_coding	232.0663519	129.5368343	-0.842354894	2.22E-07	6.47E-06	Down
ENSG00000137752.25	CASP1	protein_coding	875.0235919	392.3086183	-1.156895482	2.29E-35	1.55E-32	Down
ENSG00000137801.11	THBS1	protein_coding	6328.844181	11255.74192	0.83063661	5.59E-07	1.48E-05	Up
ENSG00000138119.17	MYOF	protein_coding	3205.201822	5480.414234	0.773818932	8.31E-06	0.000149003	Up
ENSG00000138193.17	PLCE1	protein_coding	402.3909363	722.2610569	0.844175954	6.20E-16	7.65E-14	Up
ENSG00000138650.9	PCDH10	protein_coding	117.2057525	265.845104	1.181209638	1.10E-11	7.52E-10	Up
ENSG00000138835.24	RGS3	protein_coding	218.6412032	341.6992446	0.644054701	4.27E-06	8.50E-05	Up
ENSG00000139531.13	SUOX	protein_coding	309.1513905	165.8101437	-0.895781488	5.14E-09	2.17E-07	Down
ENSG00000139915.22	MDGA2	protein_coding	87.17675705	142.4499885	0.710502762	0.00035821	0.003384791	Up
ENSG00000140522.12	RLBP1	protein_coding	364.8790086	237.2787417	-0.620417744	8.62E-07	2.14E-05	Down
ENSG00000141404.17	GNAL	protein_coding	385.3160238	192.2854313	-1.002154508	7.25E-14	6.92E-12	Down
ENSG00000141527.19	CARD14	protein_coding	173.4326189	98.30897419	-0.817516618	5.21E-06	0.000100273	Down
ENSG00000141540.11	TTYH2	protein_coding	3672.017603	2415.137029	-0.604221422	7.93E-32	3.77E-29	Down
ENSG00000143127.13	ITGA10	protein_coding	66.73974183	103.5818088	0.63398031	0.003855174	0.021908589	Up
ENSG00000143375.15	CGN	protein_coding	327.3664823	193.3943337	-0.76007309	1.35E-07	4.14E-06	Down
ENSG00000143603.19	KCNN3	protein_coding	68.84292076	151.3098734	1.135465025	8.73E-09	3.52E-07	Up
ENSG00000143702.16	CEP170	protein_coding	584.8376022	969.7880514	0.729200588	1.07E-12	8.79E-11	Up
ENSG00000143786.8	CNIH3	protein_coding	108.5653964	47.44469867	-1.19211235	2.62E-07	7.50E-06	Down
ENSG00000144218.21	AFF3	protein_coding	233.2876649	131.4299846	-0.828516307	3.54E-08	1.27E-06	Down
ENSG00000144857.15	BOC	protein_coding	498.5797234	331.4934426	-0.588810394	9.13E-09	3.66E-07	Down
ENSG00000146285.15	SCML4	protein_coding	205.6082428	81.99264041	-1.326973615	4.31E-13	3.74E-11	Down
ENSG00000147027.4	TMEM47	protein_coding	265.2109809	531.4023239	1.002128392	6.08E-19	1.05E-16	Up
ENSG00000147437.10	GNRH1	protein_coding	25.84216044	49.44243157	0.93370913	0.004769091	0.025734643	Up
ENSG00000147804.10	SLC39A4	protein_coding	386.422266	250.6159519	-0.626214986	2.78E-07	7.85E-06	Down
ENSG00000148450.13	MSRB2	protein_coding	463.2009512	291.375459	-0.669023624	6.92E-08	2.30E-06	Down
ENSG00000148735.16	PLEKHS1	protein_coding	77.00107243	245.2852644	1.67089148	2.89E-15	3.23E-13	Up
ENSG00000150551.11	LYPD1	protein_coding	29.86117173	53.99161397	0.85184528	0.007757714	0.037395563	Up
ENSG00000150687.12	PRSS23	protein_coding	1200.367471	1872.697075	0.642121539	1.30E-20	2.52E-18	Up
ENSG00000151012.13	SLC7A11	protein_coding	1772.110756	3224.753476	0.864103364	1.08E-24	3.07E-22	Up
ENSG00000151224.13	MAT1A	protein_coding	153.2358339	89.63880418	-0.770211919	0.00029342	0.002909707	Down
ENSG00000151914.22	DST	protein_coding	5126.241076	7736.784893	0.593850909	4.85E-05	0.000666872	Up
ENSG00000152128.13	TMEM163	protein_coding	498.8622529	220.4051177	-1.177255547	4.22E-24	1.15E-21	Down
ENSG00000152439.13	ZNF773	protein_coding	88.0310449	141.5113517	0.684856803	0.000491837	0.004373702	Up
ENSG00000152894.15	PTPRK	protein_coding	77.0462601	192.9666803	1.325485702	5.79E-10	2.94E-08	Up
ENSG00000152942.19	RAD17	protein_coding	123.9887198	267.7675246	1.108663543	1.29E-10	7.31E-09	Up
ENSG00000153253.20	SCN3A	protein_coding	32.73843813	61.33356638	0.907666019	0.002450798	0.015476694	Up
ENSG00000153993.14	SEMA3D	protein_coding	46.24543333	73.55155714	0.671582784	0.009896305	0.044905123	Up
ENSG00000154162.15	CDH12	protein_coding	61.08102418	121.3379222	0.992090475	7.40E-06	0.000135177	Up
ENSG00000154217.16	PITPNC1	protein_coding	1118.926112	1859.479878	0.73298496	1.49E-25	4.78E-23	Up
ENSG00000154556.20	SORBS2	protein_coding	155.3458373	327.3193357	1.075202786	6.90E-11	4.06E-09	Up
ENSG00000154639.19	CXADR	protein_coding	654.521212	392.0674401	-0.738658076	1.74E-14	1.78E-12	Down
ENSG00000154856.13	APCDD1	protein_coding	396.5576568	156.9918804	-1.335874371	1.16E-16	1.53E-14	Down
ENSG00000154864.14	PIEZO2	protein_coding	71.88852888	109.1275501	0.605931467	0.009988163	0.04520921	Up
ENSG00000155849.16	ELMO1	protein_coding	84.31107248	28.45401873	-1.56825913	4.41E-08	1.54E-06	Down
ENSG00000155850.9	SLC26A2	protein_coding	9665.138527	15749.58933	0.704530006	4.45E-37	3.36E-34	Up
ENSG00000155966.14	AFF2	protein_coding	35.16545988	63.587621	0.852761088	0.005759157	0.029745033	Up
ENSG00000156103.16	MMP16	protein_coding	3074.900034	4617.518607	0.586623215	3.71E-17	5.12E-15	Up
ENSG00000156453.14	PCDH1	protein_coding	491.358497	807.0791928	0.7158169	4.11E-14	4.02E-12	Up
ENSG00000158022.7	TRIM63	protein_coding	865.8722751	486.1897684	-0.833088804	1.10E-21	2.43E-19	Down
ENSG00000158352.18	SHROOM4	protein_coding	413.609002	626.3930116	0.599716631	1.23E-08	4.81E-07	Up
ENSG00000158747.15	NBL1	protein_coding	4542.143688	3021.529647	-0.58788895	8.14E-18	1.24E-15	Down
ENSG00000159685.11	CHCHD6	protein_coding	2778.495138	1604.71653	-0.79166379	2.76E-29	1.22E-26	Down
ENSG00000160200.18	CBS	protein_coding	104.4061231	178.1496804	0.772251505	0.000408881	0.003774651	Up
ENSG00000161249.21	DMKN	protein_coding	68.05183382	36.02113048	-0.91548171	0.001210818	0.009043779	Down
ENSG00000161970.15	RPL26	protein_coding	333.8866822	199.897193	-0.741927663	1.03E-07	3.26E-06	Down
ENSG00000162066.16	AMDHD2	protein_coding	617.9737713	405.0915871	-0.607557845	1.14E-08	4.48E-07	Down
ENSG00000162409.12	PRKAA2	protein_coding	72.32543711	111.703561	0.628203812	0.003401315	0.019866032	Up
ENSG00000162645.13	GBP2	protein_coding	54.83279145	92.84237873	0.761688356	0.002444692	0.01545277	Up
ENSG00000162733.19	DDR2	protein_coding	772.2263493	1185.132312	0.618540835	6.40E-16	7.82E-14	Up
ENSG00000162878.13	PKDCC	protein_coding	81.15352704	124.3737872	0.614396313	0.003962462	0.022334123	Up
ENSG00000162944.11	RFTN2	protein_coding	174.7604458	276.05966	0.660142157	7.82E-06	0.000141395	Up
ENSG00000163235.16	TGFA	protein_coding	526.2866094	1158.594398	1.138511728	2.09E-42	2.44E-39	Up
ENSG00000163568.16	AIM2	protein_coding	59.71365008	116.8641944	0.97216488	5.84E-05	0.000780018	Up
ENSG00000163599.18	CTLA4	protein_coding	102.8838802	64.57324837	-0.671666903	0.002849555	0.017362529	Down
ENSG00000164106.8	SCRG1	protein_coding	46.15654538	76.43149478	0.728467606	0.005270949	0.02784554	Up
ENSG00000164175.15	SLC45A2	protein_coding	4580.135189	3043.949204	-0.589218379	2.53E-27	9.03E-25	Down
ENSG00000164176.13	EDIL3	protein_coding	29.18617515	94.36016894	1.690714062	1.95E-09	8.92E-08	Up
ENSG00000164418.22	GRIK2	protein_coding	481.1266472	944.3477263	0.97297022	2.50E-23	6.28E-21	Up
ENSG00000164946.20	FREM1	protein_coding	33.6480023	73.46031981	1.130428312	0.000143294	0.001646154	Up
ENSG00000165105.10	RASEF	protein_coding	2951.464207	1752.482299	-0.752098032	1.79E-38	1.44E-35	Down
ENSG00000165238.17	WNK2	protein_coding	412.5220434	250.7625553	-0.717459118	2.86E-10	1.54E-08	Down
ENSG00000165507.9	DEPP1	protein_coding	59.03323588	127.3397325	1.11137227	3.42E-07	9.53E-06	Up
ENSG00000165626.19	BEND7	protein_coding	98.39322677	46.69885343	-1.07869313	0.000116539	0.001380388	Down
ENSG00000165948.11	IFI27L1	protein_coding	29.04200509	72.54023314	1.322661526	7.58E-06	0.000138095	Up
ENSG00000166046.11	TCP11L2	protein_coding	95.47817351	143.5962275	0.590595544	0.004589625	0.025061307	Up
ENSG00000166741.9	NNMT	protein_coding	351.675336	691.7936195	0.977426239	5.09E-21	1.05E-18	Up
ENSG00000166912.17	MTMR10	protein_coding	120.4516901	190.6011399	0.661189913	0.003405521	0.019881551	Up
ENSG00000167191.12	GPRC5B	protein_coding	4256.785674	2774.879301	-0.616975088	2.15E-27	7.86E-25	Down
ENSG00000167601.12	AXL	protein_coding	64.36208877	109.2429704	0.759910066	0.006640842	0.033255749	Up
ENSG00000168505.7	GBX2	protein_coding	251.6745049	139.7447646	-0.848519545	4.86E-08	1.68E-06	Down
ENSG00000168685.15	IL7R	protein_coding	51.63421139	121.1112492	1.227557705	1.02E-06	2.52E-05	Up
ENSG00000168772.12	CXXC4	protein_coding	46.15830576	126.9758247	1.459886671	5.89E-11	3.55E-09	Up
ENSG00000169020.10	ATP5ME	protein_coding	110.035878	61.06752154	-0.852435147	0.000278699	0.002802717	Down
ENSG00000169246.17	NPIPB3	protein_coding	107.8649578	67.3455514	-0.680248746	0.004582007	0.025051687	Down

Geneid	gene_name	gene_type	mean_norm_430	mean_norm_430_KO	log_2FoldChange	pvalue	padj	change
ENSG00000169302.16	STK32A	protein_coding	380.8029591	191.8551527	-0.986561359	3.68E-14	3.66E-12	Down
ENSG00000169306.11	IL1RAPL1	protein_coding	179.5806384	296.6512281	0.72240594	2.43E-06	5.21E-05	Up
ENSG00000169744.13	LDB2	protein_coding	234.6362791	380.7111386	0.699643556	1.24E-07	3.84E-06	Up
ENSG00000169783.13	LINGO1	protein_coding	403.9222039	259.0319078	-0.643143389	1.43E-06	3.32E-05	Down
ENSG00000169891.18	REPS2	protein_coding	126.8019022	71.45002319	-0.82375632	0.000237732	0.00246213	Down
ENSG00000170153.11	RNF150	protein_coding	430.0320642	1234.055051	1.521842927	1.91E-69	1.39E-65	Up
ENSG00000170379.21	TCAF2	protein_coding	210.8261499	351.3317582	0.738011062	2.88E-08	1.05E-06	Up
ENSG00000170396.9	ZNF804A	protein_coding	28.97099389	67.58393746	1.221401878	2.92E-05	0.000437946	Up
ENSG00000170579.19	DLGAP1	protein_coding	239.6320721	134.3460297	-0.836017604	7.69E-07	1.94E-05	Down
ENSG00000170631.15	ZNF16	protein_coding	216.8454835	348.9461499	0.685785818	8.93E-08	2.89E-06	Up
ENSG00000171224.9	FAM241B	protein_coding	122.9922731	80.06366724	-0.620753733	0.003846383	0.021868317	Down
ENSG00000171368.12	TPPP	protein_coding	764.4455342	407.1010815	-0.90888191	4.59E-22	1.07E-19	Down
ENSG00000171522.6	PTGER4	protein_coding	47.41223296	87.02176551	0.873480735	0.00050551	0.004466427	Up
ENSG00000172061.9	LRRC15	protein_coding	42.88203229	96.36168694	1.165600307	1.04E-05	0.000180486	Up
ENSG00000172260.15	NEGR1	protein_coding	11.46828177	78.86189528	2.787961996	7.31E-13	6.25E-11	Up
ENSG00000172318.6	B3GALT1	protein_coding	56.64059454	92.51807881	0.706287405	0.006007929	0.030629221	Up
ENSG00000172578.12	KLHL6	protein_coding	164.4527994	95.01967654	-0.792879024	0.000102997	0.001243326	Down
ENSG00000172817.4	CYP7B1	protein_coding	186.8406655	373.2483033	0.998058664	5.29E-12	3.88E-10	Up
ENSG00000172927.9	MYEOV	protein_coding	65.95369102	38.82305753	-0.763819099	0.008902433	0.041449935	Down
ENSG00000173114.13	LRRN3	protein_coding	56.47615506	88.33817622	0.644011521	0.007132329	0.03506592	Up
ENSG00000174473.17	GALNTL6	protein_coding	64.45058944	142.1463917	1.141901777	6.11E-08	2.06E-06	Up
ENSG00000175356.14	SCUBE2	protein_coding	220.3651264	122.4657533	-0.844342002	4.26E-06	8.50E-05	Down
ENSG00000175899.15	A2M	protein_coding	1464.307337	2564.349771	0.808631768	5.77E-32	2.85E-29	Up
ENSG00000176049.16	JAKMIP2	protein_coding	230.8762637	405.0906426	0.811213996	1.16E-11	7.91E-10	Up
ENSG00000176463.14	SLCO3A1	protein_coding	251.0155104	151.8856201	-0.726330934	6.42E-07	1.65E-05	Down
ENSG00000176771.18	NCKAP5	protein_coding	48.83147694	81.36961867	0.737308779	0.00444334	0.024449802	Up
ENSG00000178445.10	GLDC	protein_coding	421.0474652	638.9922076	0.601588082	3.88E-10	2.02E-08	Up
ENSG00000178531.6	CTXN1	protein_coding	63.04809413	38.71733969	-0.70465904	0.010455281	0.046795316	Down
ENSG00000178568.16	ERBB4	protein_coding	15.89636659	62.53271319	1.975150995	8.85E-08	2.87E-06	Up
ENSG00000179314.16	WSCD1	protein_coding	16.51710063	59.01550942	1.838030199	5.05E-07	1.35E-05	Up
ENSG00000181104.7	F2R	protein_coding	382.1025977	678.6518301	0.82824641	8.07E-18	1.24E-15	Up
ENSG00000181458.10	TMEM45A	protein_coding	570.9313376	919.1752227	0.687434694	8.11E-15	8.60E-13	Up
ENSG00000182111.9	ZNF716	protein_coding	84.48898487	131.0428176	0.633260717	0.000961991	0.007531584	Up
ENSG00000182168.16	UNC5C	protein_coding	93.94512333	329.648418	1.810446471	1.60E-28	6.61E-26	Up
ENSG00000182253.15	SYNM	protein_coding	3276.509965	5110.334316	0.641286535	3.45E-35	2.22E-32	Up
ENSG00000182256.13	GABRG3	protein_coding	109.448009	36.51008792	-1.584955912	6.75E-10	3.37E-08	Down
ENSG00000182580.3	EPHB3	protein_coding	206.4309302	332.5592335	0.689447428	1.84E-07	5.49E-06	Up
ENSG00000182612.11	TSPAN10	protein_coding	156.2270488	83.80572106	-0.896755598	2.09E-06	4.58E-05	Down
ENSG00000182667.15	NTM	protein_coding	692.0333848	1257.575322	0.861825377	1.53E-28	6.54E-26	Up
ENSG00000183054.13	RGPD6	protein_coding	37.89591855	78.52256944	1.053392048	0.000669508	0.005596749	Up
ENSG00000183098.12	GPC6	protein_coding	802.0274758	1443.702473	0.848616296	3.65E-22	8.78E-20	Up
ENSG00000183779.7	ZNF703	protein_coding	1181.604891	767.9637299	-0.620847733	6.20E-12	4.45E-10	Down
ENSG00000183873.18	SCN5A	protein_coding	103.7400649	68.44049405	-0.600732429	0.0066792	0.033401204	Down
ENSG00000184226.15	PCDH9	protein_coding	134.1805488	397.4789541	1.566599869	4.07E-28	1.58E-25	Up
ENSG00000184381.20	PLA2G6	protein_coding	744.3054102	441.6128674	-0.75279788	4.88E-16	6.14E-14	Down
ENSG00000184613.11	NELL2	protein_coding	22.26445543	78.85489115	1.830687917	9.97E-08	3.20E-06	Up
ENSG00000185304.16	RGPD2	protein_coding	25.69056738	53.06634612	1.042966622	0.005044442	0.026881346	Up
ENSG00000185442.13	FAM174B	protein_coding	131.6412082	82.10751528	-0.681169255	0.000558012	0.004838111	Down
ENSG00000185585.20	OLFML2A	protein_coding	484.6825394	929.5062619	0.939896298	9.55E-26	3.14E-23	Up
ENSG00000185614.7	INKA1	protein_coding	287.0099246	157.0499447	-0.86899966	1.76E-07	5.28E-06	Down
ENSG00000185668.8	POU3F1	protein_coding	173.6700807	108.3090178	-0.679725444	9.01E-05	0.001113525	Down
ENSG00000185737.13	NRG3	protein_coding	90.07454366	41.66118584	-1.10925286	2.71E-05	0.000412688	Down
ENSG00000186297.12	GABRA5	protein_coding	68.18340826	40.94276326	-0.736955551	0.00682225	0.033969017	Down
ENSG00000186472.20	PCLO	protein_coding	374.5344849	666.6276208	0.832167607	8.70E-15	9.15E-13	Up
ENSG00000186897.5	C1QL4	protein_coding	97.78773181	62.93497524	-0.636170069	0.006436474	0.03245298	Down
ENSG00000187135.8	VSTM2B	protein_coding	47.97113918	26.67929226	-0.849626339	0.009820505	0.044655109	Down
ENSG00000188158.17	NHS	protein_coding	42.73560605	118.8643895	1.474181306	4.31E-08	1.51E-06	Up
ENSG00000188488.14	SERPINA5	protein_coding	68.53104126	115.1120187	0.746421021	0.001253845	0.009268055	Up
ENSG00000188501.12	LCTL	protein_coding	36.45753325	62.80805736	0.784166067	0.007110216	0.034997428	Up
ENSG00000188994.14	ZNF292	protein_coding	831.1106858	1274.392753	0.616699004	1.21E-10	6.89E-09	Up
ENSG00000189184.12	PCDH18	protein_coding	76.8759072	129.2952777	0.749391607	0.000163162	0.001825369	Up
ENSG00000196154.12	S100A4	protein_coding	1250.012436	1923.007373	0.621424765	3.02E-20	5.71E-18	Up
ENSG00000196159.14	FAT4	protein_coding	120.3013616	249.3150147	1.051624473	6.69E-12	4.77E-10	Up
ENSG00000196376.11	SLC35F1	protein_coding	523.4187829	1156.516641	1.143298579	1.84E-42	2.35E-39	Up
ENSG00000196646.12	ZNF136	protein_coding	158.6884509	260.979525	0.716090914	1.09E-06	2.66E-05	Up
ENSG00000196660.12	SLC30A10	protein_coding	104.5642619	54.21869009	-0.949584701	3.79E-05	0.000542485	Down
ENSG00000197692.5	CBX3P7	processed_pseudogene	104.4546087	58.50636959	-0.833635948	0.000483079	0.004313754	Down
ENSG00000198223.18	CSF2RA	protein_coding	195.182615	109.8855353	-0.827003611	1.37E-06	3.22E-05	Down
ENSG00000198373.13	WWP2	protein_coding	2820.137313	4533.030829	0.684726994	7.74E-44	1.42E-40	Up
ENSG00000198743.7	SLC5A3	protein_coding	3947.237943	7240.816677	0.875356455	2.42E-10	1.32E-08	Up
ENSG00000198868.3	MTND4LP30	processed_pseudogene	50.84185987	27.70672896	-0.87253343	0.009173568	0.0423406	Down
ENSG00000200087.1	SNORA73B	snoRNA	89.07760113	55.92427841	-0.670990397	0.004631396	0.025235698	Down
ENSG00000204356.14	NELFE	protein_coding	354.8742113	145.8363439	-1.282922973	0.00113756	0.008639099	Down
ENSG00000204469.13	PRRC2A	protein_coding	130.6391046	50.45576617	-1.372041077	0.00686024	0.03408076	Down
ENSG00000204839.9	MROH6	protein_coding	54.8441225	30.0267027	-0.868726958	0.005018966	0.026790088	Down
ENSG00000204920.11	ZNF155	protein_coding	39.94483494	66.17311887	0.727408398	0.011350697	0.04942387	Up
ENSG00000204956.6	PCDHGA1	protein_coding	31.30547045	58.51375214	0.90284369	0.004001265	0.022479963	Up
ENSG00000205213.14	LGR4	protein_coding	992.9242569	1537.319398	0.631070502	5.99E-21	1.22E-18	Up
ENSG00000205786.8	LINC01531	lncRNA	148.0344082	56.24081737	-1.396092912	6.67E-10	3.34E-08	Down
ENSG00000206077.14	ZDHHC11B	protein_coding	358.9377557	176.3418778	-1.024817902	2.10E-15	2.39E-13	Down
ENSG00000213949.10	ITGA1	protein_coding	367.9119126	592.5085296	0.687928858	1.00E-12	8.29E-11	Up
ENSG00000218336.9	TENM3	protein_coding	1602.476669	2722.00965	0.764684358	4.66E-32	2.49E-29	Up
ENSG00000223382.6	LINC01778	lncRNA	33.15292928	62.27667606	0.908768828	0.002069332	0.013674045	Up
ENSG00000224397.9	PELATON	lncRNA	457.6642595	273.1602968	-0.744144647	5.86E-10	2.96E-08	Down
ENSG00000224660.2	SH3BP5-AS1	lncRNA	718.6093447	459.1432069	-0.64650037	1.44E-11	9.59E-10	Down
ENSG00000224934.6	GOT1-DT	lncRNA	85.56172394	133.2919726	0.640848434	0.001151988	0.008685261	Up
ENSG00000226508.1	LINC01918	lncRNA	439.6849612	213.8781487	-1.038461406	3.61E-15	3.96E-13	Down
ENSG00000227203.3	SUB1P1	processed_pseudogene	71.61064251	34.19927121	-1.065110004	0.000376139	0.003523076	Down
ENSG00000228253.1	MT-ATP8	protein_coding	11492.62467	6491.539168	-0.824107854	8.38E-09	3.39E-07	Down
ENSG00000229400.1	CNIH3-AS1	lncRNA	51.20926649	25.1087612	-1.023930975	0.003476974	0.020179345	Down
ENSG00000230082.1	PRRT3-AS1	lncRNA	112.5297579	71.3841979	-0.654589939	0.002007039	0.013351124	Down
ENSG00000231312.8	MAP4K3-DT	lncRNA	208.9154902	132.6684786	-0.654031023	2.51E-05	0.00038378	Down
ENSG00000232310.10	LINC03002	lncRNA	57.1040671	33.00320114	-0.787990475	0.010191067	0.045852655	Down

Geneid	gene_name	gene_type	mean_norm_430	mean_norm_430_KO	log$_2$FoldChange	pvalue	padj	change
ENSG00000233527.10	ZNF529-AS1	lncRNA	25.70516838	48.90989031	0.932346354	0.008261541	0.039147747	Up
ENSG00000233539.5	ENSG00000233539	lncRNA	83.01836683	48.44395588	-0.782870866	0.003749134	0.021497517	Down
ENSG00000234323.10	LINC01505	lncRNA	77.51867103	118.315687	0.609295543	0.00232884	0.014904579	Up
ENSG00000234362.7	LINC01914	lncRNA	51.48047066	82.40088894	0.682754037	0.010135846	0.045701247	Up
ENSG00000235172.9	LINC01366	lncRNA	131.125615	74.68232263	-0.811118357	6.67E-05	0.000871032	Down
ENSG00000237289.10	CKMT1B	protein_coding	132.4878165	80.16647533	-0.72805329	0.004071669	0.022785722	Down
ENSG00000237499.7	WAKMAR2	lncRNA	113.0577017	71.31837261	-0.666372311	0.003182098	0.018816322	Down
ENSG00000237638.3	LINC02245	lncRNA	87.75266272	48.74064239	-0.849508253	0.000650633	0.005456713	Down
ENSG00000240476.1	LINC00973	lncRNA	86.73570157	50.35527798	-0.787261322	0.002134538	0.013988962	Down
ENSG00000240695.1	DNAJC8P2	processed_pseudogene	78.37446846	48.21687976	-0.703454092	0.007613665	0.036839573	Down
ENSG00000241081.1	RPL22P2	processed_pseudogene	60.72670899	35.19190272	-0.78740969	0.007937944	0.038035733	Down
ENSG00000241945.8	PWP2	protein_coding	60.12046744	28.64853639	-1.066535658	0.001542771	0.01083571	Down
ENSG00000242083.2	RPL7AP31	processed_pseudogene	59.4853774	30.30483901	-0.970668857	0.001718511	0.01179879	Down
ENSG00000243244.7	STON1	protein_coding	129.8954779	227.4823275	0.808577039	3.31E-06	6.85E-05	Up
ENSG00000246859.3	STARD4-AS1	lncRNA	104.9549629	189.8946872	0.856753222	6.15E-07	1.59E-05	Up
ENSG00000248923.1	MTND5P11	processed_pseudogene	57.59435478	33.43179905	-0.78798374	0.009829218	0.044678899	Down
ENSG00000248964.7	ENSG00000248964	lncRNA	78.45241265	49.26833242	-0.669059645	0.008713187	0.040761072	Down
ENSG00000249992.2	TMEM158	protein_coding	484.1384686	236.6409095	-1.032863115	2.38E-17	3.36E-15	Down
ENSG00000250378.4	ENSG00000250378	lncRNA	70.18700049	34.079309	-1.041129295	0.000455696	0.004115055	Down
ENSG00000250479.9	CHCHD10	protein_coding	1351.168468	885.0390071	-0.611073124	4.94E-11	3.03E-09	Down
ENSG00000251191.8	LINC00589	lncRNA	329.8106342	190.4215266	-0.793446768	4.33E-09	1.84E-07	Down
ENSG00000254595.1	ENSG00000254595	processed_pseudogene	27.68722661	49.86109091	0.853457881	0.010604641	0.047216285	Up
ENSG00000254876.5	SUGT1P4-STRA6LP	lncRNA	52.30026959	84.72519319	0.6932456	0.007033495	0.034686137	Up
ENSG00000255248.11	MIR100HG	lncRNA	215.6394096	342.8089039	0.667657637	1.14E-07	3.57E-06	Up
ENSG00000256894.1	ENSG00000256894	lncRNA	186.5386016	115.0013073	-0.697621332	1.91E-05	0.000302508	Down
ENSG00000258645.2	HSPE1P2	processed_pseudogene	172.2702231	105.7359413	-0.70349862	0.000100568	0.001220894	Down
ENSG00000258791.9	LINC00520	lncRNA	1774.22349	1137.752565	-0.641456278	1.51E-20	2.88E-18	Down
ENSG00000260877.3	ENSG00000260877	lncRNA	88.01682539	56.58603275	-0.637077571	0.007295493	0.035663148	Down
ENSG00000260920.2	ENSG00000260920	lncRNA	31.71341589	58.72989673	0.889315197	0.003908677	0.022114703	Up
ENSG00000261036.2	ENSG00000261036	lncRNA	38.61863195	65.43004108	0.76316866	0.007429611	0.036139789	Up
ENSG00000261115.7	TMEM178B	protein_coding	138.0351206	415.1080527	1.589377209	4.85E-32	2.49E-29	Up
ENSG00000261616.1	TTC23-AS1	lncRNA	42.70865444	77.00761984	0.849843889	0.001113434	0.008486217	Up
ENSG00000266835.7	GAPLINC	lncRNA	80.80350138	46.19196034	-0.806203907	0.002460095	0.01551422	Down
ENSG00000269821.2	KCNQ1OT1	lncRNA	538.0130319	857.1325722	0.671988955	0.003033978	0.01818403	Up
ENSG00000270647.7	TAF15	protein_coding	963.6923134	2077.131842	1.107968555	0.002224024	0.014400198	Up
ENSG00000273145.1	ENSG00000273145	lncRNA	101.8390844	65.48571637	-0.638733821	0.009369191	0.042999089	Down
ENSG00000274525.1	ENSG00000274525	unprocessed_pseudogene	45.12132019	72.50949763	0.683927445	0.007894341	0.037925941	Up
ENSG00000276107.1	THBS1-IT1	lncRNA	39.54582787	69.80898261	0.815988254	0.004899727	0.026384096	Up
ENSG00000276168.1	RN7SL1	misc_RNA	1465.254072	209.4440566	-2.806553704	0.004844694	0.026098703	Down
ENSG00000276476.4	LINC00540	lncRNA	99.25670378	52.28271279	-0.92502302	0.000192566	0.002087709	Down
ENSG00000276644.5	DACH1	protein_coding	90.28859684	204.2629769	1.177402452	3.52E-12	2.67E-10	Up
ENSG00000277586.4	NEFL	protein_coding	1120.942534	628.5729139	-0.834830514	3.70E-22	8.78E-20	Down
ENSG00000279198.2	ENSG00000279198	TEC	61.64559882	26.69199828	-1.212216739	0.000104177	0.001250509	Down
ENSG00000279518.1	ENSG00000279518	TEC	41.41834145	72.98075554	0.82057501	0.003475821	0.020179345	Up
ENSG00000279970.1	ENSG00000279970	TEC	80.75880951	39.50676874	-1.027346733	0.001066084	0.00818181	Down
ENSG00000285925.1	ENSG00000285925	lncRNA	56.93005696	24.35385276	-1.226242397	0.000589747	0.00504173	Down
ENSG00000286451.1	ENSG00000286451	lncRNA	73.76671088	39.13029715	-0.912050802	0.000669935	0.005596749	Down
ENSG00000288728.2	ENSG00000288728	lncRNA	44.65105113	74.5148492	0.740794294	0.006398297	0.032298564	Up

Supplementary figures

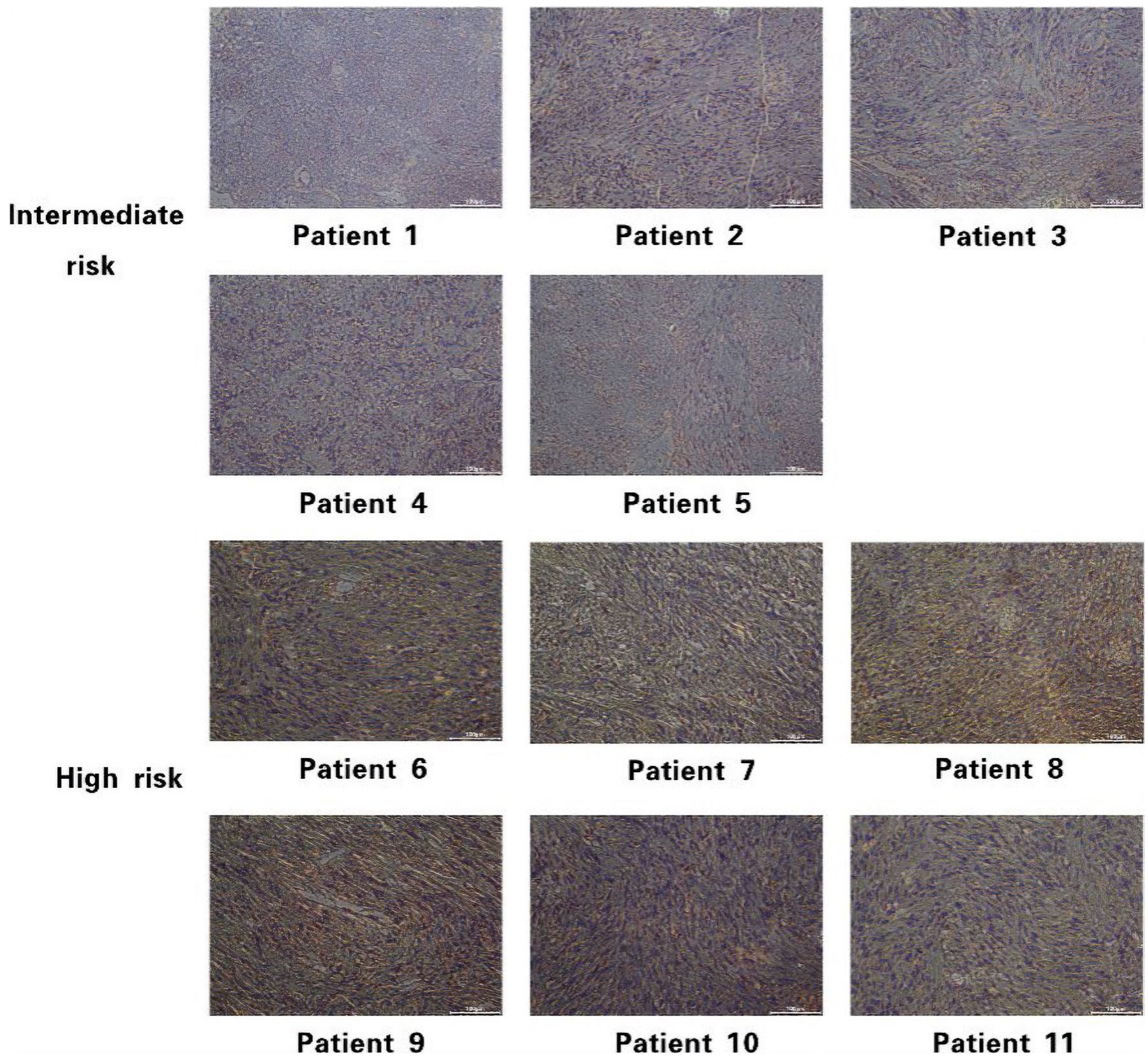

SUPPLEMENTARY FIGURE S1. Immunohistochemistry (IHC) analysis of EPHA3 in paraffin-embedded sections of tumors from 11 GIST patients, including 5 intermediate-risk and 6 high-risk cases. Original magnification, 200×; scale bar = 100 μm.

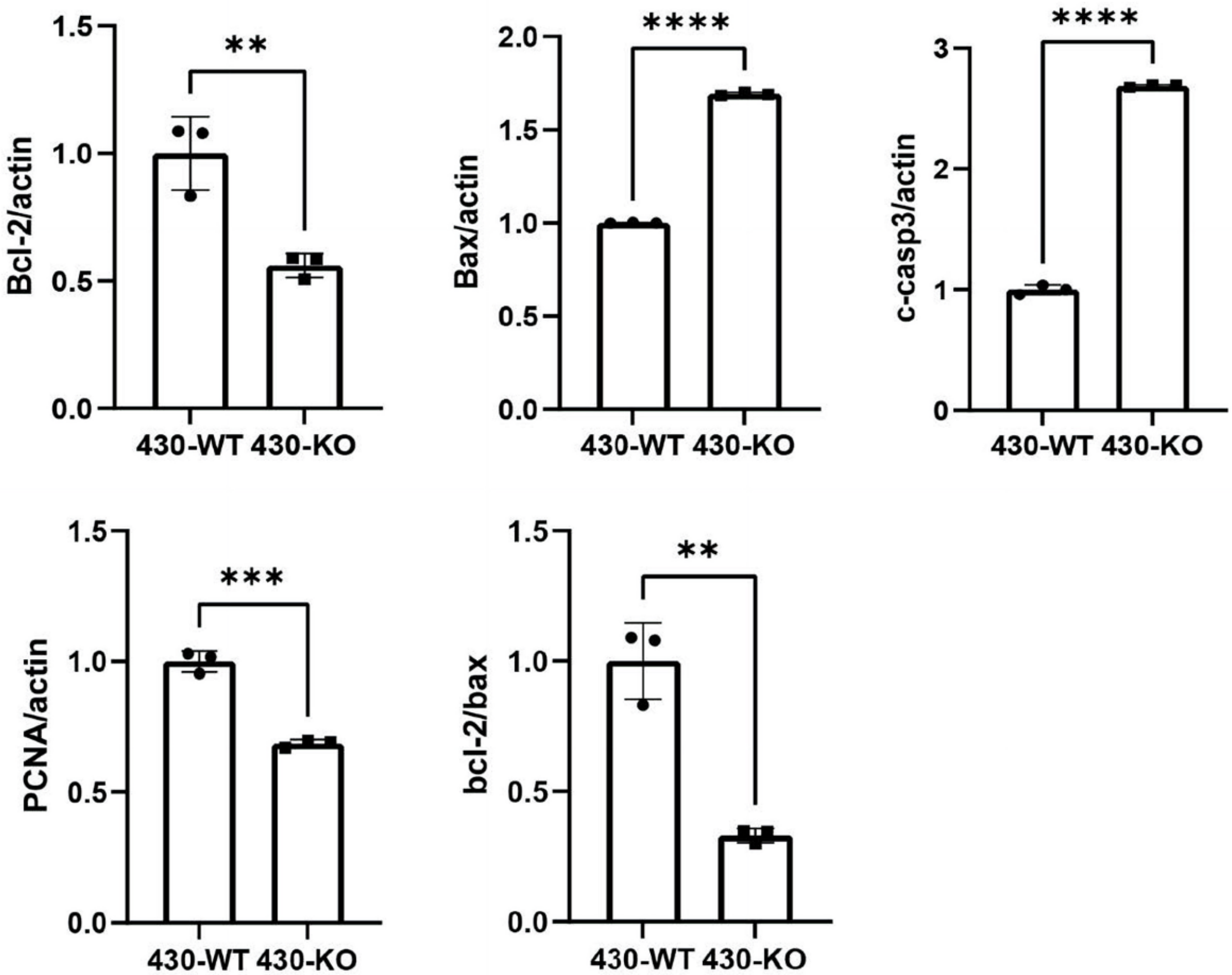

SUPPLEMENTARY FIGURE S2. Band intensities from Western blots (Figure 2A) were quantified using ImageJ (NIH). The relative expression levels of Bcl-2, Bax, PCNA, and cleaved caspase-3 were calculated by normalizing each target protein to β-actin. The Bcl-2/Bax ratio was calculated from the corresponding band intensities. Data from three independent experiments were averaged, and values were normalized to the mean of the 430-WT group, which was set to 1. Data are shown as mean ± SD from three independent experiments. Statistical significance was determined by paired t-test. **p* < 0.05, ***p* < 0.01, ****p* < 0.001, *****p* < 0.0001.

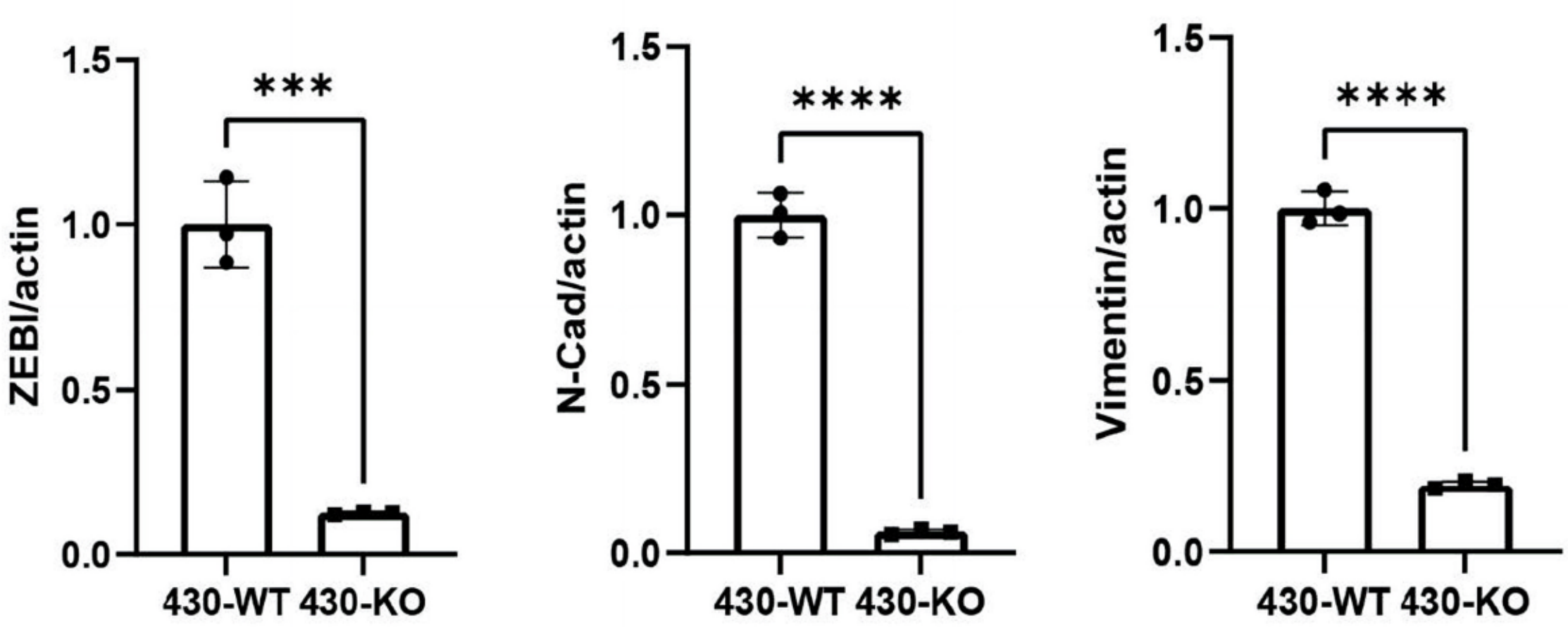

SUPPLEMENTARY FIGURE S3. Band intensities from Western blots (Figure 3D) were quantified using ImageJ (NIH). The relative expression levels of ZEB1, N-cadherin, and Vimentin were calculated by normalizing each target protein to β-actin. Data from three independent experiments were averaged, and values were normalized to the mean of the 430-WT group, which was set to 1. Data are shown as mean ± SD from three independent experiments. Statistical significance was determined by paired t-test. **p* < 0.05, ***p* < 0.01, ****p* < 0.001, *****p* < 0.0001.

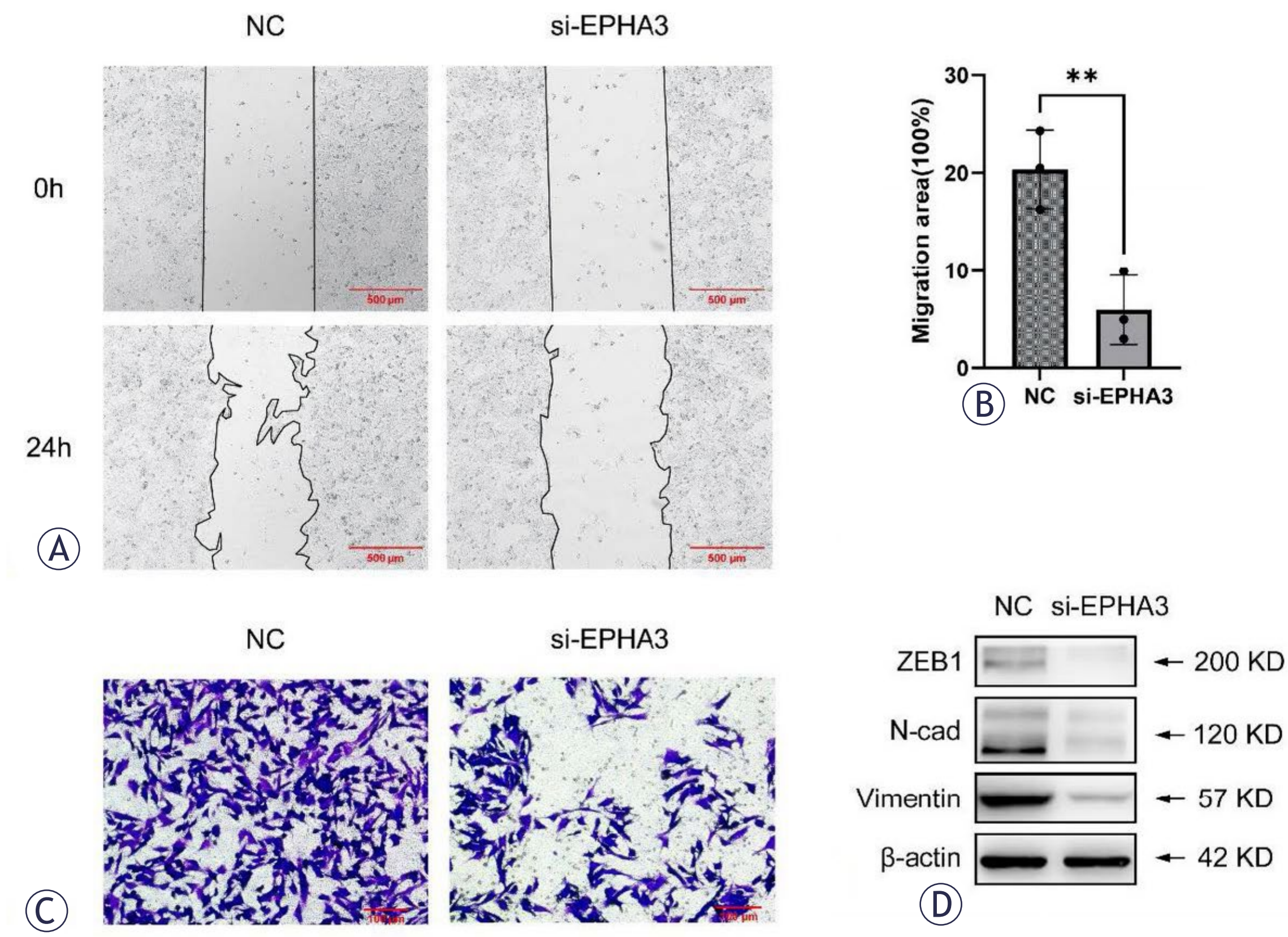

SUPPLEMENTARY FIGURE S4. EPHA3 knockdown inhibits the migration of GIST-430 cells. **(A-B)** Wound-healing assay showing the migratory capacity of GIST-430 cells transfected with negative control siRNA (NC) or si-EPHA3 at 0 and 24 h. Scale bar = 500 μm. (C) Transwell migration assay showing the migratory ability of GIST-430 cells transfected with NC or si-EPHA3. **(D)** Representative Western blot images showing the expression of EPHA3, ZEB1, N-cadherin, and Vimentin in GIST-430 cells transfected with NC or si-EPHA3, with β-actin as the loading control. Data are presented as the mean ± SD from three independent experiments, ***p* < *0.01*.
